# Mechanistic Insight Into the Polymorph‐Dependent Photosalient Effect of a White Light or Two‐Photon Activated Photoreactive Molecular Salt

**DOI:** 10.1002/anie.5518257

**Published:** 2026-05-23

**Authors:** Emanuela Santagata, Francesco Ambrosio, Amedeo Capobianco, Andrea Peluso, Giorgia Rizzi, Stijn Van Cleuvenbergen, Yovan de Coene, Koen Clays, Paolo Pio Mazzeo, Roberto Centore, Fabio Borbone

**Affiliations:** ^1^ Department of Chemical Sciences University of Naples Federico II Naples Italy; ^2^ Department of Science University of Basilicata Potenza Italy; ^3^ Department of Chemistry and Biology ‘Adolfo Zambelli’/DCB University of Salerno Fisciano (SA) Italy; ^4^ Department of Chemistry Katholieke Universiteit Leuven Leuven Belgium; ^5^ Department of Chemistry Life Sciences and Environmental Sustainability University of Parma Parma Italy

**Keywords:** cycloaddition, photosalient effect, polymorphism, reversible photoreaction, two‐photon absorption, visible light activation

## Abstract

The photosalient effect (PE) provides a visually striking demonstration of the direct transduction of light energy into macroscopic mechanical work. However, establishing a predictive, atomic‐level understanding of how the underlying crystal packing governs the accumulation and release of macroscopic stress remains a challenge. Here, we report two photoreactive polymorphs of a stilbene‐type molecular salt (OHT‐T^I^ and OHT‐T^II^) that undergo the identical topochemical [2 + 2] photocycloaddition but exhibit divergently different mechanical behaviors: while OHT‐T^II^ reacts statically, OHT‐T^I^ displays a violent, explosive photosalient actuation. By combining single‐crystal x‐ray diffraction with advanced periodic density functional theory (DFT) calculations, we reveal the mechanistic origin of this phase‐dependent selectivity. We demonstrate that the specific shell‐like packing of OHT‐T^I^ completely frustrates the structural relaxation of the newly formed cyclobutane dimer, leading to accumulation of elastic strain. Conversely, the uninterrupted stacking in OHT‐T^II^ allows for the unhindered dissipation of this strain. Furthermore, the pronounced push–pull character of the chromophore effectively overcomes the traditional reliance on UV light, enabling this actuation to be cleanly triggered by low‐energy visible light and, unprecedentedly, via near‐infrared two‐photon absorption. Supported by complete thermal reversibility, this study provides a predictive structural and computational blueprint for the rational design of next‐generation dynamic materials.

## Graphical abstract

Photocycloaddition in stilbene‐type salt polymorphs exhibit distinct light‐driven responses: OHT‐T^I^ shows a violent photosalient effect under visible and NIR (two‐photon) irradiation, whereas OHT‐T^II^ reacts statically. Packing and computational analysis reveals that shell‐like structures in OHT‐T^I^ frustrate structural relaxation, accumulating explosive strain. The system enables a reversible light‐and‐thermal‐driven process for next‐generation smart materials.

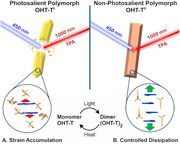

## 1 Introduction

The high relevance of research on photosalient compounds lies in their potential applications in the development of smart materials that can convert light energy into mechanical motion [1, 2]. These compounds exhibit the remarkable phenomenon of crystals undergoing rapid and significant shape changes when exposed to light, ultimately causing movements such as bending and twisting, or even leaping [3, 4, 5, 6, 7, 8]. This ability to directly convert light into mechanical work offers immense prospects in various fields. The rapid and forceful expansion or sudden explosion of these crystals upon light exposure could be used for actuators in micro‐ or nanoscale devices, sensors to detect the presence of light or other stimuli and trigger a signal [9], or even in artificial muscles, and micro‐machines [10, 11]. The propensity to cause crystal disintegration can be exploited for applications requiring a single actuation event, such as electrical fuses [12]. Drug delivery applications can also be imagined, for instance by encapsulating drugs in hollow crystals and using light‐induced bursting as the release mechanism [13], possibly taking advantage of near‐infrared light and its deep tissue penetration.

The macroscopic‐scale mechanical motions exhibited by photosalient crystals in response to light are the result of structural changes occurring at the molecular level, usually induced by a topochemical reaction such as [2 + 2] cycloaddition or photodimerization [14]. According to the topochemical postulate (Schmidt rules), the distance between the centers of the reacting double bonds (C═C) in adjacent molecules should be less than 4.2 Å, while the reacting double bonds must be aligned parallel to each other [15]. However, these rules are not absolute and several factors, such as steric hindrance, limited molecular motion, and the existence of alternative reaction pathways, can prevent the reaction from occurring even if the criteria are met. Potentially, the reaction might also proceed when these criteria are not met [16, 17, 18, 19, 20, 21, 22]. Understanding the precise relationship between crystal structure, molecular packing, and the photosalient effect is crucial for designing materials with tailored responses [16, 23]. In this context, polymorphism provides an unprecedented opportunity to decouple the intrinsic reactivity of a chromophore from the mechanical constraints imposed by its crystalline environment. Here, we present a combined crystallographic and computational investigation into a stilbene‐type ionic compound with tosylate as a counterion (OHT‐T), to provide a fundamental mechanistic rationale for the polymorph‐dependent photosalient effect.

We selectively crystallized two distinct polymorphs (OHT‐T^I^ and OHT‐T^II^), both of which appear to respect the Schmidt rule for [2 + 2] cycloaddition. However, only OHT‐T^I^ exhibited a photosalient effect, manifested as a rapid light‐driven burst, while the second polymorph underwent the cycloaddition reaction statically, without any visible phenomenon falling within the definition of photosalient effect. To unravel the origin of this divergent macroscopic behavior, we employed advanced periodic density functional theory (DFT) calculations alongside single‐crystal x‐ray diffraction (SCXRD). This combined approach reveals that the photosalient explosion in OHT‐T^I^ originates from frustrated packing strain: its specific shell‐like crystal packing completely prevents the structural relaxation of the newly formed dimer, leading to a critical accumulation of elastic energy. Conversely, the uninterrupted stacking in OHT‐T^II^ allows for the unhindered dissipation of this strain.

In addition, our system successfully overcomes a major practical limitation of most photosalient materials relying on photodimerization. With regard to photosalient explosions, the majority of reported compounds rely on UV‐driven photoactivation. However, visible light actuation would be a more practical option for real‐world applications. In a few instances, 1‐D polymers and metal–organic frameworks undergo additional activation by sunlight, but the observed effects are relatively slow and sluggish [24, 25]. In our system, the increased push‐pull character of the conjugated backbone improves the matching of the absorption band of the photoactive cation with the visible spectrum, enabling a rapid and clean photoreaction triggered by white or monochromatic visible light. Furthermore, we show for the first time that the photosalient effect can be successfully induced by multiphoton absorption using near‐infrared light [26]. Finally, it was observed that the photoinduced cycloaddition product can be thermally decomposed back to the initial photoreactive polymorph with high yield, giving rise to a reversible switch between two species with selective sensitivity to light and heat.

## 2 Results and Discussion

**FIGURE 1:**
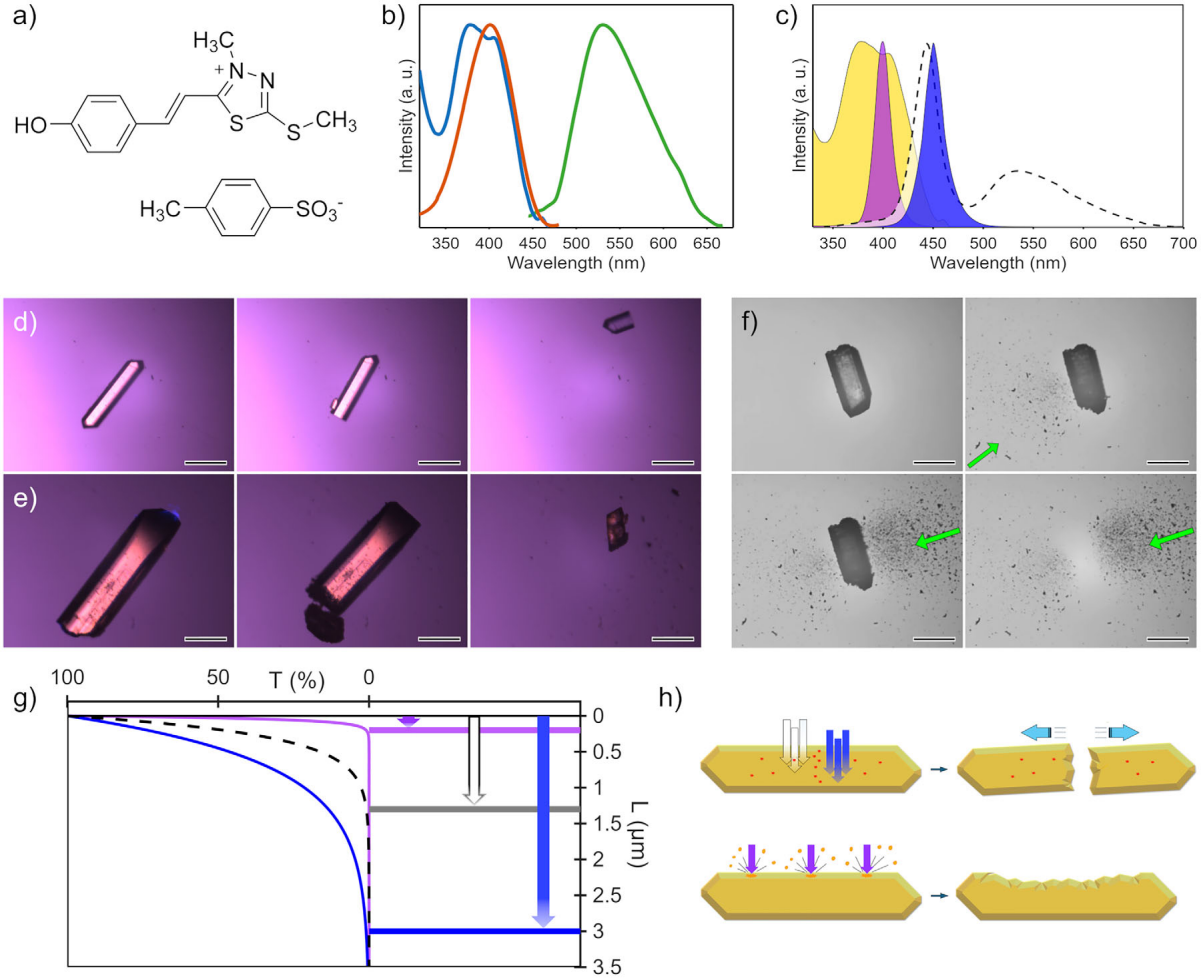
(a) Chemical structure of OHT‐T; (b) absorption spectrum of OHT‐T in 1.90·10^−5^ M acetonitrile solution (orange line), excitation and emission spectra of OHT‐T^I^ (blue and green line, respectively); (c) Excitation spectrum of OHT‐T^I^ (yellow area) compared with the emission spectra of violet, blue and white LEDs used in the experiments; (d) PE of OHT‐T^I^ crystal under white LED illumination; (e) PE of OHT‐T^I^ crystal under blue LED illumination; (f) PE of OHT‐T^I^ crystal under violet LED illumination (green arrows indicate the direction of light incidence); (g) Decay of *λ*_max_ transmittance across the crystal for the violet (purple line), white (dashed line) and blue (blue line) LEDs and their penetration depths; (h) Schematic representation of the PE under different wavelength illumination. Scale bar is 50 µm.

OHT‐T is the tosylate salt of the (E)‐2‐(4‐hydroxystyryl)‐3‐methyl‐5‐(methylthio)‐1,3,4‐thiadiazol‐3‐ium cation (Figure 1a), which is synthesized according to the procedure reported in the Supporting Information. We managed to selectively crystallize two distinct polymorphs depending on the synthetic pathways employed. Condensation of 4‐hydroxybenzaldehyde with the iodide salt of cation T^+^ (2, Scheme S1), followed by metathesis in water with sodium tosylate, produced orange, needle‐shaped single crystals of the polymorph I (OHT‐T^I^) by slow cooling of the water solution. When the condensation was directly conducted with the tosylate salt of T^+^, selective crystallization of reddish prisms of a second polymorph (OHT‐T^II^) was achieved (see Supporting Information). Crystals of both polymorphs underwent [2 + 2] photocycloaddition reaction when exposed to the UV lamp. Surprisingly, even pure white light from an LED or selected single‐color LEDs in the visible range were able to activate the reaction. While the two polymorphs may exhibit different quantum yields for photocycloaddition, the visible‐activated reaction was found to be rapid and complete, yielding pure photoproducts with high yield and no detectable byproducts. The ^1^H NMR spectra of OHT‐T^I^ (and similarly for OHT‐T^II^) recorded at various time intervals demonstrate complete conversion of the monomer after 20 h of white light illumination (Figure S1). The reaction proceeded as well when the crystals were illuminated with a blue LED (450 nm). This activation by pure white/blue LEDs was enabled by the absorption properties of the OHT^+^ cation (Figure 1a), a stilbene type molecule with a strong electronic push–pull character. This is due to the rather strong electron withdrawing effect of the *N*‐methylated thiadiazole ring and the electron donor effect of the phenolic OH. The UV‐visible spectrum of OHT‐T in solution shows a band with a maximum at 401 nm and a cutoff around 450 nm (Figure 1b). This is mostly reproduced by the excitation spectrum of OHT‐T^I^ (Figure 1b) and OHT‐T^II^ powders (Figure S2), recorded by monitoring the intensity of fluorescence maximum. When exposed to light, the crystals of OHT‐T^I^ exhibited a photosalient effect, resulting in their fragmentation into separate pieces that were propelled in opposite directions. This behavior was reproduced both under a white LED (Figure 1d; Movie 1) and a blue LED (Figure 1e; Movie 2).

This type of motility was predominant and appeared to be largely independent of crystal size for both light sources. No motions such as rolling or translation were observed. The dynamics underwent a substantial shift when a violet LED (395 nm) was used for illumination. In this case, it has been observed that the crystals undergo an erosion process that starts from the surface. This process involves the ejection of numerous small fragments that are progressively projected in the direction opposite to that from which the radiation originates (Figure 1f, Movie 3). When the erosion reaches a critical depth, the fragmentation affects the inner part of the crystal, leading to the typical burst observed under visible light. The observed discrepancies in behavior are attributed to the different penetration depths of the radiation wavelengths used in the experiments, as the emission spectra of the three LEDs, all of which have similar luminous flux, have significantly different overlap with the absorption spectrum of OHT‐T^I^ (Figure 1c). Therefore, it is reasonable to assume that blue and white radiation would be able to reach deeper into the bulk of the crystal and induce photoproduct formation in a diffuse manner within a large volume, until a critical threshold of defects causing fragmentation is reached. In contrast, violet radiation has poor penetration ability due to its strong absorption by the surface layers of the crystal, where it is able to induce the maximum concentration of photoproduct (and defects). This results in the immediate ejection of numerous small fragments (Figure 1h). We simulated the penetration depth of the radiation at the wavelengths of emission maxima of the three LEDs (450, 444, and 400 nm for the blue, white and violet LEDs, respectively), by calculating the transmittance as a function of the radiation path through the crystal, using the Lambert–Beer law (see Supporting Information) [27]. The light path corresponding to a decrease in transmittance to a negligible value (below 1%) can be defined as the penetration depth, corresponding to approximately 3.0 µm, 1.3 µm, and 200 nm for the blue, white and violet LEDs, respectively (Figure 1g). These values support the hypothesis that most of the photons of blue and white LEDs are expected to pass through the crystal for longer distances (Figure 1h). In the case of the violet LED, all photons are absorbed by a layer measuring a few hundred nanometers below the crystal surface, producing a completely different macroscopic effect, which may eventually result in the fragmentation and jump of the residual crystal core.

**FIGURE 2:**
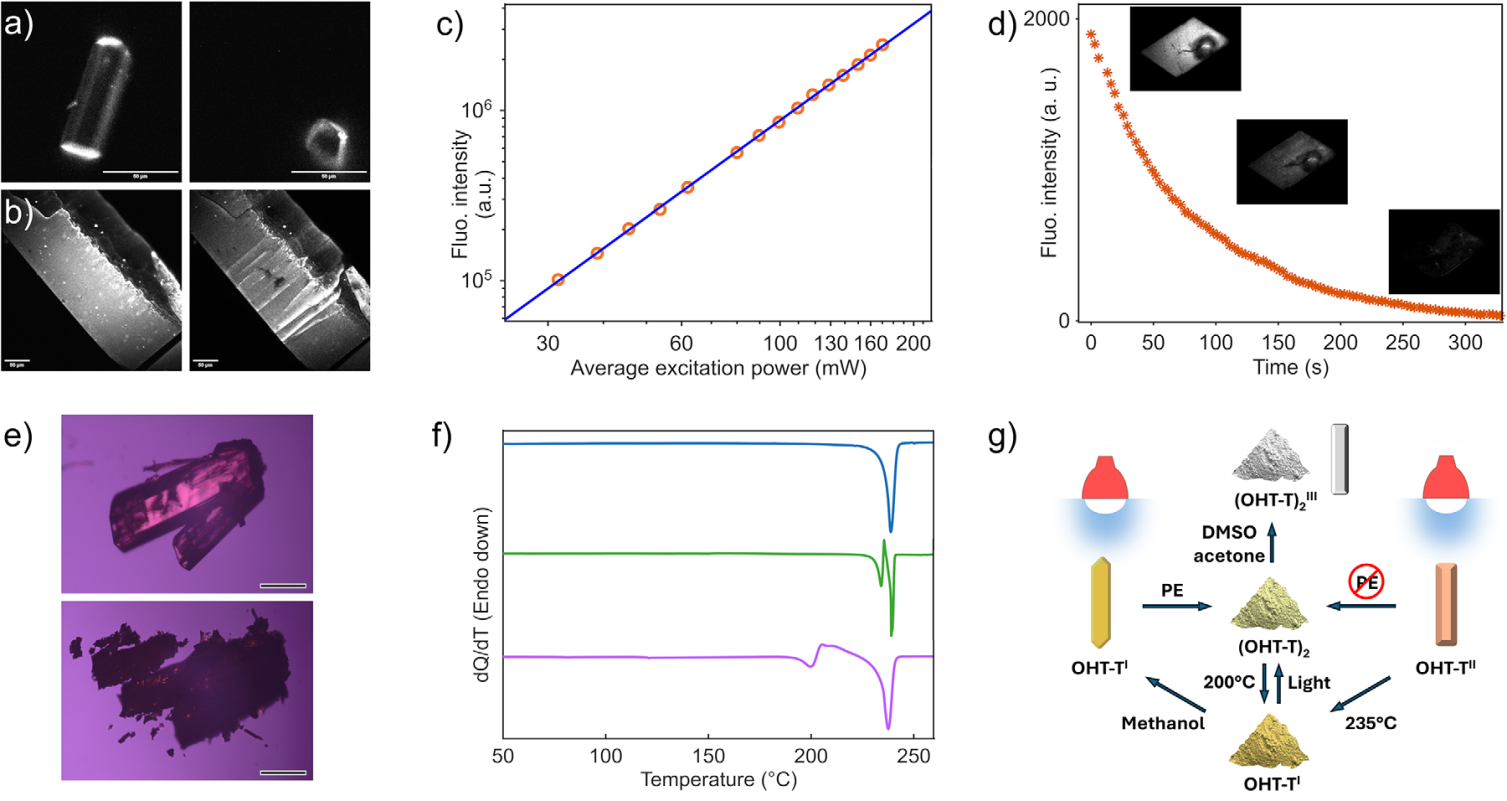
(a) NLO microscopy imaging of the two‐photon absorption induced photosalient effect on OHT‐T^I^; (b) NLO microscopy imaging of the two‐photon absorption induced defects on OHT‐T^I^ crystals; (c) Multiphoton fluorescence intensity of OHT‐T^I^ as a function of the fundamental wavelength (1030 nm) excitation power; (d) Decay of the MPF of an OHT‐T^I^ crystal during illumination with 1030 nm laser pulses; (e) Photoreaction of OHT‐T^II^ crystals under white LED illumination; (f) DSC diagrams of OHT‐T^I^ (blue line), OHT‐T^II^ (green line) and (OHT‐T)_2_^I^ (purple line); (g) Scheme depicting the photo‐ and thermal conversion of OHT‐T and (OHT‐T)_2_ polymorphs. Scale bar is 50 µm.

We expanded our use of longer wavelength light to induce the PE in OHT‐T^I^, and we present here for the first time the PE induced by multi‐photon absorption. In our experiments, we irradiated the crystals with a femtosecond pulsed laser set at 1000 nm and imaged them with scanning NLO microscopy [28, 29]. We recorded the outgoing signal of multi‐photon fluorescence (MPF) and second harmonic generation (SHG) when present. Although there is no one‐photon absorption by OHT‐T at this wavelength, the same PE was observed as the one induced by blue/white light. This consisted of the typical splitting/jumping phenomenon (Figure 2a; Movie 4). The effect required a longer time to occur than the irradiation with visible light. This is due to the multi‐photon nature of the absorption and the intrinsic confinement of the nonlinear effect. Based on the objective (NA 0.8), the lateral resolution (*xy* plane) is 400 nm, but due to quadratic (two photon) or cubic (three photon) dependence, this can be considered an upper limit [30]. The high beam penetration and confined nature of the interaction are also consistent with the explosive nature of the observed PE. This localized illumination allowed the process to be slowed down significantly compared to the use of single photon illumination. Consequently, the change in crystal morphology prior to the occurrence of the photosalient effect could be evidenced in real time through the use of NLO microscopy. During the initial minutes of exposure to the 1000 nm laser pulses, the initially smooth surface of the crystal begins to show areas of highly regular linear discontinuities oriented perpendicular to the crystal's main axis (Figure 2b). These irregularities are consistent with the observed direction of splitting and jumping [17]. By plotting the intensity of the recorded MPF versus the power of the fundamental incident wavelength on logarithmic scales, we obtained a linear correlation with a slope of 2 (Figure 2c), indicating that the phenomenon is driven by two‐photon absorption (TPA) [31].

While the absolute 2PA cross‐section was not determined, the clear power‐law dependence is sufficient to establish the non‐linear nature of the phenomenon. We were also able to monitor the MPF of OHT‐T^I^ in real time during the irradiation process. We observed a progressive decay of fluorescence due to the photoreaction that produced the non‐fluorescent photoproduct (Figures 2d and S3). When OHT‐T^II^ crystals were exposed to the same conditions, they retained their initial shape and position and underwent a gradual deterioration of their crystal structure, without any additional motion‐related effects. As illustrated in Figure 2e, the original shape of the two single crystals is preserved after the photoreaction, with only slight fragmentation. The different photomechanical responses of the two polymorphs were clearly observed after prolonged exposure of a crystal specimen over a glass substrate. While the crystals of OHT‐T^II^ remained largely immobile, those of OHT‐T^I^ gradually disintegrated and disseminated across the surface, propelling the resulting debris several centimeters away. The complete photoconversion of both polymorphs OHT‐T^I^ and OHT‐T^II^ resulted in the formation of a fine powder of the two photoproducts named (OHT‐T)_2_^I^ and (OHT‐T)_2_^II^, respectively. These phases produced the PXRD patterns reported in Figure S4. The DSC analysis of OHT‐T^II^ crystals showed a melting point of approximately 235°C, followed by rapid recrystallization into OHT‐T^I^, which melts at 239°C (Figure 2f). Interestingly, the thermal analysis of (OHT‐T)_2_^I^ showed decomposition at about 200°C, followed by recrystallization. The decomposition product was identified as OHT‐T^I^, exhibiting a melting peak indistinguishable from the initial crystals (Figure 2f). This behavior was consistent in our experimental setup, demonstrating that thermal reconversion of the dimer to the initial photosalient phase is possible. This provides a mechanism for exploiting PE in a repeatable manner on the same starting material. Recrystallisation of (OHT‐T)_2_^I^ powder or crystals from anhydrous dimethyl sulfoxide and acetone yielded a white powder showing no variation in the NMR spectra, but a different PXRD pattern (Figure S4). This new phase was also successfully grown as single crystals and named (OHT‐T)_2_^III^. The conversion process between phases is outlined in Figure 2g.

**FIGURE 3:**
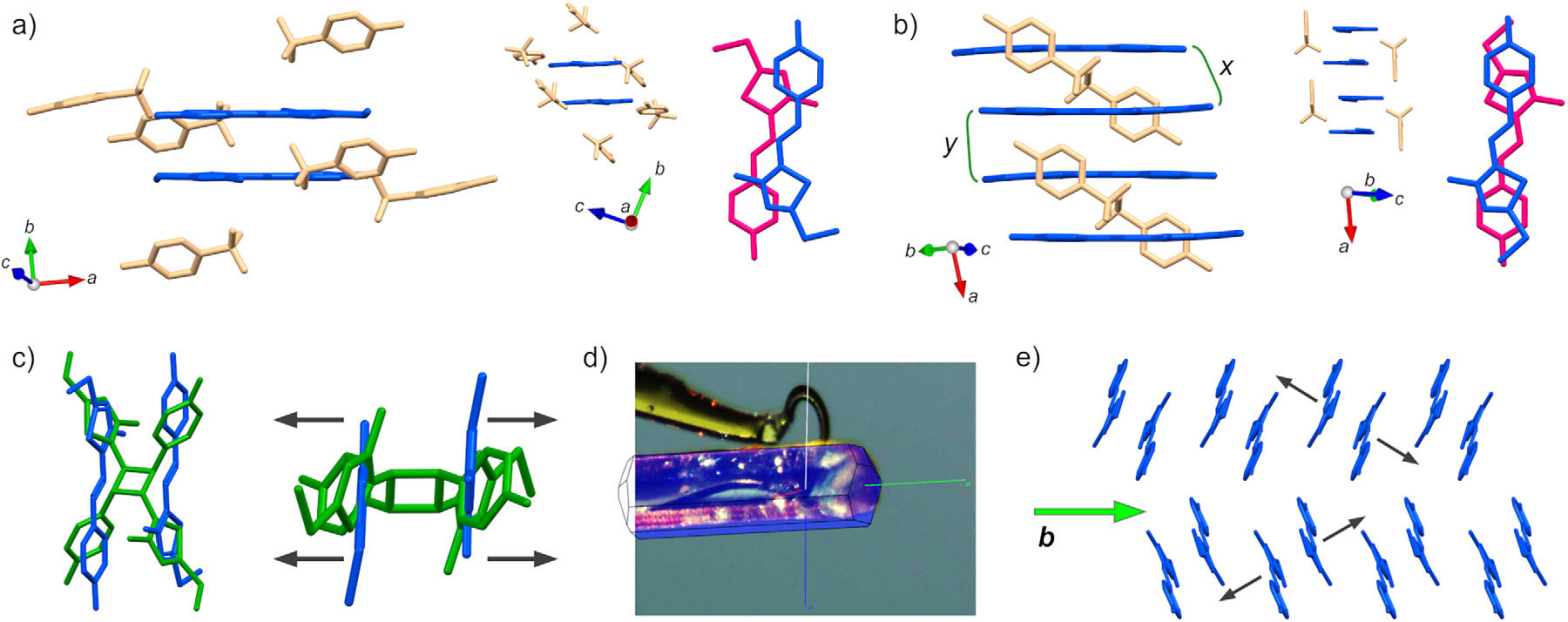
Detail of crystal packing of OHT‐T^I^ (a) and OHT‐T^II^ (b); (c) Overlay of OHT‐T^I^ (blue molecules) and (OHT‐T)_2_^III^ (green molecule) dimers, with expansion directions indicated by black arrows; (d) Cell axes orientation in the crystal of OHT‐T^I^ (same color code as the figures); (e) Detail of crystal packing of OHT‐T^I^ along *b* axis (green arrow) with expansion directions indicated by black arrows.

The solid‐state structure of both OHT‐T polymorphs and of (OHT‐T)_2_^III^ was solved by x‐ray diffraction analysis (Table S1, CCDC numbers 2526788, 2526605, and 2526789 for OHT‐T^I^, OHT‐T^II^, and (OHT‐T)_2_^III^, respectively). Comparison of calculated and experimental PXRD patterns are reported in Figures S5, S6, and S7 for OHT‐T^I^, OHT‐T^II^, and (OHT‐T)_2_^III^. Polymorph OHT‐T^I^ crystallizes in the monoclinic system, space group P2_1_/c, while polymorph OHT‐T^II^ in the triclinic system, space group $P\bar{1}$. In both centrosymmetric polymorphs, there is one ion pair in the asymmetric unit and the OHT^+^ cation adopts a highly planar conformation (Figure 3a,b). While electrostatic interactions between the benzothiadiazolium cations and the tosylate anions represent the strongest type of interaction within the crystal structure, strong hydrogen bonds are also present between the OH group of OHT^+^ and the sulfonate group of the tosylate anion, in both OHT‐T^I^ and OHT‐T^II^ (OH⋯O─S distance of 1.70 and 1.80 Å, respectively). Weak CH⋯O hydrogen bonds also occur between the sulfonate group and the phenyl ring, CH═CH and the methyl of tosylate, with a richer pattern in OHT‐T^II^ as compared to OHT‐T^I^ (Figure S8). The C═C bonds are parallel and the distance between their centroids is 3.749 Å for OHT‐T^I^, 3.634 Å and 3.702 Å for dimers *x* and *y* of OHT‐T^II^, respectively (Figure 3b). Therefore, it can be concluded that both polymorphs fulfil the conditions of the Schmidt rules for photochemical [2 + 2] cycloaddition reaction, in accordance with the experimental behavior. To gain insight into the crystal structure of the (OHT‐T^I^)_2_ photoproduct, we irradiated multiple crystals of OHT‐T^I^ until they fractured. However, no measurable changes in cell parameters were detected by SCXRD. This indicates that the photosalient effect in OHT‐T^I^ is initiated by a minimal amount of photoproduct, remaining below the threshold for monitoring single‐crystal‐to‐single‐crystal reactivity. A closer inspection of the crystal packing of OHT‐T polymorphs reveals a markedly different arrangement of cations, which may account for the different macroscopic responsiveness of the crystals of the two phases, as also suggested by the theoretical analysis (vide infra). In the OHT‐T^I^ structure, pairs of cations exhibit an antiparallel arrangement within centrosymmetric dimers, facilitated by π‐stacking interactions. This arrangement is further encapsulated within a shell of tosylate anions, which act as a barrier towards further stacking, effectively isolating the dimers (Figure 3a). In contrast, uninterrupted stacking of antiparallel molecules occurs along the *a* axis of the cell in OHT‐T^II^ (Figure 3b), thus extending the interaction throughout the crystal. In OHT‐T^I^, crystal breaking occurs along surfaces perpendicular to the crystal axis *b* (Figure 3c–e). This process results in the propulsion of debris along this axis direction. This is due to the expansion occurring in that direction as a result of the [2 + 2] cycloaddition. However, this expansion is frustrated in OHT‐T^I^, due to shell‐like packing creating a strain that is released at some point, giving rise to the PE. In contrast, the expansion in OHT‐T^II^ is not hindered by the packing arrangement and can propagate through the entire crystal thickness. This enables the crystal to relieve stress during the reaction, thereby effectively preventing the accumulation of energy required to trigger the PE.

**FIGURE 4:**
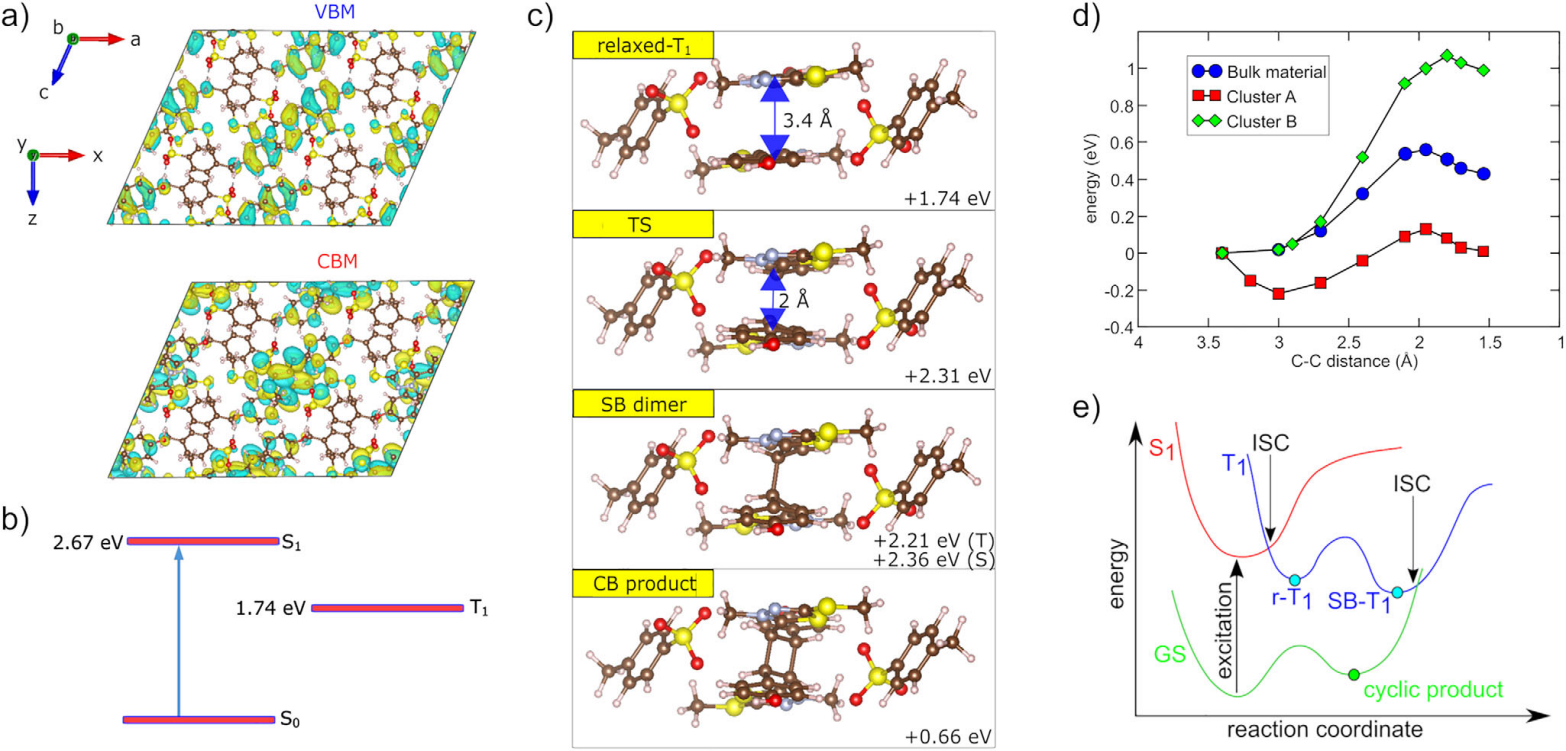
(a) Isodensity representation of the valence band maximum and conduction band minimum for OHT‐T^I^. In the representation, the *b* lattice vector points outwards. C atoms in brown, H in white, N in blue, O in red, S in yellow; (b) Jablonski diagram of OHT‐T^I^; (c) Structural representation of relaxed triplet state (r‐T1), transition state (TS), singly bound dimer (SB), and cyclobutane (CB) product in bulk OHT‐T^I^. For clarity of representation, only the stilbene units involved in the reaction and the closest counter‐ions are depicted in the figure. Same color codes as in (a). The total energy of each structural configuration is also reported, as referred to that of the supercell of the pristine material in the ground state. For the SB dimer, we include the energy for both the triplet (T) and the singlet (S) excited states; (d) Total energy of the supercell as a function of the decreasing intermolecular C─C distance for two stilbene units forming a bound dimer (see text and Supporting Information for definition of cluster A and B); (e) Proposed reaction mechanism of [2 + 2]‐photocycloaddition in crystalline OHT‐T phases.

As cited above, this interpretation was also suggested by the results of a computational analysis, performed to investigate in detail this different behavior of the two phases. Our initial focus was on the phase displaying photosalient effect. The periodic DFT calculations provided a fundamental gap of 3.13 eV for OHT‐T^I^. Figure 4a shows that the electronic densities corresponding to both the valence band maximum (VBM) and the conduction band minimum (CBM) are localized on the cationic units, resembling the molecular orbitals of the isolated molecule, with negligible contribution of the counter‐ions. Next, we calculated the optical band gap at the TD‐DFT level, obtaining a value of 2.67 eV. This indicates the formation of a Frenkel exciton with a significant binding energy of 0.46 eV, which is characteristic of organic molecular crystals with a low dielectric constant. An analysis of the excited states indicated that the excitation primarily responsible for the optical gap exhibited a pronounced HOMO–LUMO character. Furthermore, we calculated the energetics of the lowest lying triplet exciton, which was found to be 2.11 eV above the ground state, if we consider the nuclear coordinates fixed to those of the ground state, and at 1.74 eV, when fully relaxing the system (c.f. Jablonski diagram in Figure 4b). This indicates a sizable difference (up to 0.93 eV) with the singlet excitation.

As well‐known, [2 + 2] photo‐cycloaddition reactions are initiated by a rapid intersystem crossing (ISC) between singlet and triplet excited states [15, 32, 33]. Following previous studies, we assume that T_1_ state is rapidly populated upon excitation to S_1_ and initiated our analysis of the reaction mechanism from the optimized structure of T_1_. Upon structural relaxation, two stilbene molecules were found to become closer to each other. Specifically, the distance between the carbon atoms of the ethylene moieties decreased from 3.7 Å in the ground state to 3.4 Å (Figure 4c), with an energy gain of 0.37 eV. Inspection of the spin wavefunction revealed a spin state that was localized on one of the two molecules but protruded partially towards the nearest neighbor (Figure S9). In other words, we observed a localized state that can evolve towards the formation of the product. This result suggests that the formation of a bond between the two molecules may imply the crossing of an energy barrier. We have therefore calculated a reaction pathway for the formation of a single C─C bond in T_1_ by performing a series of constrained structural relaxations, at fixed values of a single intermolecular C─C distance. The resulting energy profile is shown in Figure 4d, blue points. Figure 4e summarizes schematically the reaction path suggested by theoretical calculations: upon excitation to S_1_, the system can undergo rapid ISC to T_1_. As T_1_ is 0.7 eV lower in energy than S_1_, it is reasonable to assume that ISC leads to populating high vibrational states of T_1_. These states have sufficient energy to reach a nuclear configuration in which a single C─C bond is formed between two stilbene molecules (SB‐T_1_ in Figure 4e). Indeed, the energy of TS is estimated to be 2.3 eV above the ground state, well below the minimum energy point of S_1_. The formation of a second C─C bond and, consequently, of the closed‐shell cyclic product is spin‐forbidden in the triplet state. However, in the SB nuclear configuration the total energy of S_0_ is very close to the T_1_ one (ca. 0.15 eV below it, Figure 4c), thus making a second ISC (T_1_ → S_0_) plausible. Once in S_0_, the system rapidly evolves towards the cyclic product (Figure 4c, CB product). This process is accompanied by a substantial energy gain of 1.70 eV, with respect to the single‐bonded intermediate. However, due to the constraints imposed by steric hindrances and intermolecular hydrogen bonds within the host crystal, the dimer may undergo significant distortion from its equilibrium structure, to partially accommodate the change from sp^2^ to sp^3^ hybridization of the involved carbon atoms.

**FIGURE 5:**
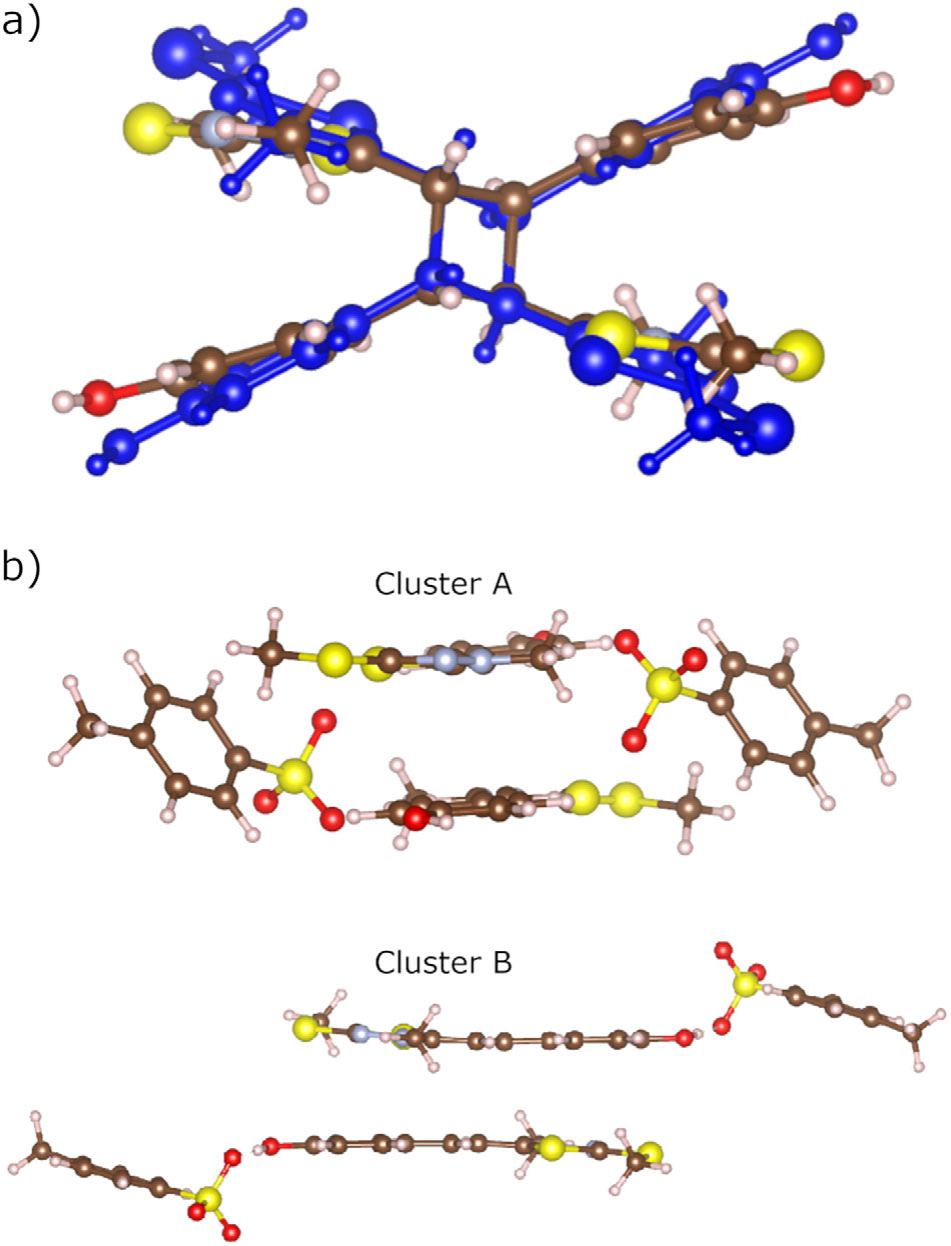
(a) Comparison between the structural features of cyclobutane derivative as resulting from the [2 + 2] photocycloaddition in solid OHT‐T^I^ phase (atoms with regular color code: C atoms in brown, H in white, N in blue, O in red, S in yellow) and after optimization of the isolated molecule (all atoms in blue); (b) Stick & ball representation of cluster A and cluster B of OHT‐T^I^. C atoms in brown, H in white, N in blue, O in red, S in yellow.

To verify this assumption, we extracted the product from the supercell and fully relaxed the isolated system, which underwent noticeable structural changes. As illustrated in Figure 5a, which shows a comparison of the structures both before and after relaxation, the “butterfly structure” of the formed cyclobutane derivative assumes its most favorable configuration. Specifically, the thiadiazole and phenol rings, no longer constrained in a strained configuration by intermolecular interactions, exhibit notable relaxation. This also produces a sizable stabilization of 0.40 eV. Therefore, as the reaction swiftly proceeds inside the crystal each formed dimer product contributes to the accumulation of a sizable strain that is eventually released. We propose that such an energy release, associated with a clearly directional relaxation motion, can be connected with the experimentally observed photosalient effect. In order to gain a more in‐depth understanding of the origin of the energy barrier for the formation of the SB configuration, we have analyzed the bond formation process by extracting a cluster consisting of two stilbene molecules and (a) two closest counter‐ions from the supercell optimized in the triplet state or (b) two counter‐ions forming hydrogen bonds with the phenol moieties of the stilbene cations, named cluster A and cluster B, respectively (Figure 5b). In the calculations, the positions of the counter‐ions were kept fixed to those of the supercell. For cluster A, we observed a noticeably different energy profile. The equilibrium distance between stilbene units shifted towards a lower distance of 3 Å. This has resulted in a reduced uphill step of only 0.3 eV, leading to products that are nearly isoenergetic with the reactants. In stark contrast, the reaction energetics for cluster B were much more similar to those observed in the solid state. These results indicate that the uphill pathway associated with bond formation in the solid is mainly due to a partial disruption of the cohesive interactions in the solid as the stilbene cations get closer to each other. In particular, the destabilization of the hydrogen bonds between the phenol moieties and the tosylate anions appears to be a key factor, while non‐directional van der Waals interactions provide a minor but still significant contribution.

Additional validation of our interpretation was provided when we modelled the OHT‐T^II^ phase. As noted above, this phase is reactive towards the [2 + 2]‐photocycloaddition but does not show any photosalient behavior upon illumination. For this system, we evaluated optoelectronic properties very similar to those calculated for the photosalient OHT‐T^I^ phase. All the computed energies were within 0.1 eV of the respective values calculated for the photosalient phase (Figure S10). The energetics of the reaction mechanism (Figure S11) also align with that of the OHT‐T^I^ phase, except for one key difference. In contrast with the result achieved for the OHT‐T^I^ phase, the final reaction product in the OHT‐T^II^ lattice was found to be in its fully relaxed structure (i.e., the blue structure in Figure 5a), without any distortion which could produce further release of energy, causing crystal scission. This outcome is consistent with the prior hypotheses, due to the unique arrangements of cations and anions in the OHT‐T^II^ lattice. These configurations allow for relatively unconstrained relaxation of the product along the *a* crystallographic direction. It is important to note that the energy of the final product is still above that of the initial ground state. This is due to the energy gain associated with the full relaxation of the dimer being partially compensated by the weakening of intermolecular interactions. In summary, the observed phenomenon prevents the accumulation of large stresses in the lattice. This fully explains the lack of photosalient behavior for the OHT‐T^II^ phase.

## 3 Conclusion

The study focused on the polymorphic photoreactive salt OHT‐T, which was successfully crystallized into two distinct phases, OHT‐T^I^ and OHT‐T^II^. Both polymorphs fulfilled the conditions of the Schmidt rules for the [2 + 2] cycloaddition reaction. We observed a polymorph‐dependent photosalient effect (PE), where OHT‐T^I^ exhibits a rapid, light‐driven burst, primarily consisting of splitting and jumping, while OHT‐T^II^ undergoes cycloaddition without exhibiting any macroscopic evidence of it, except for slight fragmentation. In comparison to other reported photosalient compounds that rely on [2 + 2] photo‐cycloaddition, OHT‐T^I^ demonstrated a notably efficient visible light activation of the reaction, including pure white light and blue LEDs (450 nm), facilitated by the strong push–pull character of the OHT^+^ cation, which optimizes the alignment of the absorption band with the visible spectrum. Experimental characterizations using different light sources (LEDs) demonstrated that the macroscopic PE behavior of OHT‐T^I^ is intrinsically linked to the penetration depth of radiation. Blue and white light, which have a deep penetration, induces the formation of diffuse photoproduct that leads to the typical burst, while more superficially absorbed violet light causes immediate surface erosion.

Furthermore, the photosalient effect was successfully induced by multi‐photon absorption (TPA) using near‐infrared light (1000 nm), which to our knowledge represents a novelty. Two‐photon absorption was confirmed by NLO microscopy measurements showing a slope of 2 in the logarithmic plot of multi‐photon fluorescence (MPF) intensity versus excitation power. Real‐time monitoring via NLO microscopy during NIR irradiation revealed the formation of highly regular linear discontinuities (defects) preceding the splitting/jumping phenomenon. Thermal characterization revealed the presence of a reversible cycle. The non‐fluorescent photoproduct, (OHT‐T)_2_, undergoes thermal decomposition at about 200°C followed by recrystallisation back into the photosalient phase OHT‐T^I^, demonstrating the potential for repeatable actuation. Structural analysis via x‐ray diffraction revealed and computational analysis confirmed that the distinctive mechanical response arises from the differences in crystal packing. In OHT‐T^I^, the antiparallel dimers are encapsulated by tosylate anions, thereby preventing the expansion associated with dimerization and forcing the cyclobutane product into a strained configuration. The experimentally observed PE is linked to the sizable energy release (0.40 eV stabilization) that is associated with the directional relaxation motion of the cyclobutane derivative when the crystal accumulated strain is released. In contrast, the uninterrupted stacking in OHT‐T^II^ allows the dimer to fully relax along one crystallographic direction, thereby preventing the accumulation of large stresses and thus explaining the absence of the PE. These findings present a rare molecular salt capable of PE activation by white light and NIR irradiation, providing essential insight into the role of lattice strain and packing frustration in light‐to‐motion conversion.

## Author Contributions

**Emanuela Santagata**: investigation, writing–review and editing. **Francesco Ambrosio**: writing–review and editing, conceptualization, investigation, writing–original draft, resources. **Amedeo Capobianco**: conceptualization, writing–review and editing, resources, writing–original draft, investigation. **Andrea Peluso**: writing–review and editing, conceptualization, resources, writing–original draft, investigation. **Giorgia Rizzi**: investigation, writing–review and editing. **Stijn Van Cleuvenbergen**: resources, writing–review and editing. **Yovan de Coene**: investigation, writing–review and editing. **Koen Clays**: writing–review and editing, resources. **Paolo Pio Mazzeo**: investigation, writing–review and editing, resources. **Roberto Centore**: conceptualization, writing–review and editing, resources, funding acquisition, investigation. **Fabio Borbone**: resources, investigation, supervision, writing–review and editing, writing–original draft, conceptualization.

## Conflicts of Interest

The authors declare no conflicts of interest.

## Supporting information




**Supporting File 1**:: anie72811‐sup‐0001‐SuppMat.docx.

**Supporting File 2**: anie72811‐sup‐0002‐MovieS1‐S4.zip.

**Supporting File 3**: anie72811‐sup‐0003‐DataFile.zip.

## Data Availability

The data that supports the findings of this study are available in the supporting information of this article
