# Supplementary material for: Mechanistic Insight Into the Polymorph‐Dependent Photosalient Effect of a White Light or Two‐Photon Activated Photoreactive Molecular Salt
Source: Angew Chem Int Ed Engl. 2026 May 23;65(30):e5518257. doi: 10.1002/anie.5518257 (PMC13383077; doi:10.1002/anie.5518257)
Supplement: Supplementary file 1 — Supporting File 1:: anie72811‐sup‐0001‐SuppMat.docx. [file ANIE-65-e5518257-s003.docx]

Supporting Information

Mechanistic Insight into the Polymorph-Dependent Photosalient Effect of a White Light or Two-Photon Activated Photoreactive Molecular Salt

Emanuela Santagata^1^, Francesco Ambrosio^2^, Amedeo Capobianco^3^, Andrea Peluso^3^, Giorgia Rizzi^4^, Stijn Van Cleuvenbergen^4^, Yovan de Coene^4^, Koen Clays^4^, Paolo Pio Mazzeo^5^, Roberto Centore^1^, Fabio Borbone^1^

^1^Department of Chemical Sciences, University of Naples Federico II, Via Cinthia, I-80126 Naples, Italy,

^2^ Department of Science, University of Basilicata, Via dell'Ateneo Lucano 10 - 85100 Potenza, Italy

^3^ Department of Chemistry and Biology ‘Adolfo Zambelli’/DCB, University of Salerno, Via Giovanni Paolo II, 132 - 84084 Fisciano (SA), Italy

^4^ Department of Chemistry Katholieke Universiteit Leuven, Leuven 3000, Belgium

^5^ Department of Chemistry, Life Sciences and Environmental Sustainability, University of Parma, Viale delle Scienze, 17A - Campus, I-43124 Parma, Italy

Experimental

All the reagents were commercially available and used without further purification. ^1^H and ^13^C NMR spectra were recorded on Varian Inova 500 (500 MHz) and Bruker Ascend 400 (400 MHz) spectrometers. DSC diagrams were recorded on a METTLER TOLEDO DSC3+ calorimeter. UV-Visible spectra were recorded with a JASCO V-750 spectrometer.

*X-ray diffraction analysis.* WAXS powder diffraction profiles were collected on powder samples with an Empyrean automatic powder diffractometer, using Cu k_α_ radiation (λ = 0.15418 nm). The profiles were recorded by a continuous scan of the diffraction angle 2θ in the interval 5-40° at a speed of 0.05252° s^−1^. Selected crystals of OHT-T^I^ and (OHT-T)_2_^III^ were mounted on a Bruker D8 Venture diffractometer equipped with a kappa goniometer and an Oxford Cryostream. Data collection was performed using a μ3 Micro-focused Mo K_α_ radiation (λ = 0.71073 Å) and a Photon III detector, further. Lorentz polarization and absorption corrections were applied for all of the experiments. Data reduction was carried out using APEX v5 software. The structure was solved by direct methods using SHELXT.^1^ The structure of OHT-T^I^ was refined with the aspherical atom refinement algorithm to achieve a more accurate structure model. Transferable aspherical atom model (TAAM)^2,3^ refinement was used by means of the DiscambMATTS2tsc software as implement in the non-spherical atoms tool in Olex2^4^ (NoSpherA2)^5^. The program built upon the new version of DiSCaMB^6^ library computes non-spherical atomic scattering factors from multipolar model parametrized using MATTS databank,^7^ while the structure of (OHT-T)_2_^III^ was refined by the full-matrix least-squares method on F^2^ using SHELXL.^1^ Selected crystals of OHT-T^II^ were mounted on a Bruker-Nonius KappaCCD diffractometer equipped with Oxford Cryostream apparatus (graphite monochromated Mo K_α_ radiation, λ = 0.71073 Å, CCD rotation images, thick slices, *ϕ* and *ω* scans to fill asymmetric unit). Reduction of data and semiempirical absorption correction were done using SADABS program. The structures were solved by direct methods (SIR97 program)^8^ and refined by the full-matrix least-squares method on F^2^ using SHELXL-2016 program^9^ with the aid of the program Olex2.^4^ H atoms bonded to C were generated stereochemically and refined by the riding model, those bonded to O and N were found in difference Fourier maps and their coordinates were refined. For all H atoms, U_iso_(H) equal to 1.2 U_eq_ or 1.5 U_eq_ (C_methyl_) of the carrier atom was used. Crystal data and structure refinement details are reported in Table S1. The figures were generated using Mercury.^10^ All crystal data were deposited at Cambridge Crystallographic Data Centre with assigned number CCDC 2526788 (*OHT-T^I^*), 2526605 (*OHT-T^II^*), 2526789 (*(OHT-T)_2_^III^*). These data can be obtained free of charge from www.ccdc.cam.ac.uk/data_request/cif.

*NLO microscopy and spectroscopy*. NLO spectra were recorded with a self-built wide-field microscope operating in transmission mode. The crystal was illuminated with a femtosecond pulsed IR laser light at 1030 nm (500 kHz, Pharos, Light Conversion, London, UK), whose intensity was controlled by rotating a zero-order half-wave plate (HWP) installed on a rotation stage (Thorlabs, PRM-Z8) and combined with a Glan-Taylor polarizer selecting for S-polarized light. The beam was focused on the sample with a long focal length lens (f = 5 cm), while the light emitted was collected underneath it by an objective (20x, 0.50 NA, CFI Plan Fluor, Nikon, Tokyo, Japan). Immediately after, an IR filter removed the laser light from the optical path. Then, a 20 cm tube lens (Mitutoyo, Japan) imaged the light into the opening of the spectrometer (Andor, Kymera 328i, Oxford, UK) coupled with the I-CCD camera (Andor, istar 340, Oxford, UK). The spectrometer could be used for spectroscopy by switching from a mirror to a grating (150 l mm^-1^ groove density; blaze = 500 nm). For the scanning microscopy setup, a mode-locked femtosecond pulsed Ti:Sapphire laser (80 MHz, 120 fs; Spectra-Physics, Insight DS+, Santa Clara, CA, USA) set at 1000 nm was used. A rotatable achromatic half-wave plate (HWP; Newport, IORP52-4, Darmstadt, GE) was used in combination with a Glan-Laser polarizer (POL; Thorlabs, GLIO) to tune to the intensity of the fundamental beam. The light is guided into the microscope (BX61WI, Olympus, Tokyo, Japan) through a series of mirrors (Thorlabs, FIO-03-M01). Inside the microscope, two Galvano scanners scan the light beam over the sample to scan the XY-plane. The laser is polarized in the X-direction, and pure linear polarization is ensured by a Glas Laser polarizer. Subsequently, an achromatic half-wave plate (Thorlabs, SAHWP05M-1700) is mounted on a rotation stage, enabling control over the polarization state. A water immersion objective (40 x, 0.80 NA, 3.5 mm WD, Nikon, Tokyo, Japan) was used. A condenser (OEM) with a numerical aperture of 0.9 was positioned after the sample to collimate the transmitted beam. The beam was then redirected by a mirror towards an analyzer (Thorlabs, WP25M) mounted on a rotation stage, enabling detection based on polarization. Two infrared filters are used to eliminate the fundamental beam, after which dichroic mirror (T525lxpr) (Chroma, Bellows Falls, VT, USA) separated the SHG and the fluorescence. The generated light was collected by photomultiplier tubes (PMT; Hamamatsu, R3896, Tokyo, Japan), each preceded by a bandpass filter (Chroma ET500/20m) to filter SHG. These were connected to a computer with software used to operate the shutter and HWPs. The setup is illustrated in Figure S12 with the detector operating in a transmission mode. The generation of SHG signals is contingent upon the concentration of a substantial number of photons at a singular point, due to the quadratic dependence of photon density. This phenomenon manifests exclusively at the laser's focal point, yielding a highly resolved three-dimensional (3D) image exploiting this inherent confocal effect. Videos were assembled from sequential images.

*Computational details*. All calculations based on density functional theory (DFT) are carried out with the freely available CP2K/QUICKSTEP suite of programs.^11^ In this code, atomic basis sets are combined with an auxiliary plane-wave basis set to re-expand the electron density. In particular, we use the MOLOPT double-zeta polarized basis set and a cutoff of 800 Ry for the plane waves.^12^ Core electrons are treated with the analytical Goedecker-Teter-Hutter pseudopotentials.^13^ To properly calculate the energetics involved in the [2+2]-photocycloaddition in OHT-T^I^ and OHT-T^II^, we employ a piece-wise linear functional belonging to the PBE0 family,^14–17^ which has found to be successful in the simulation of the structural and electronic properties of a variety of solid and liquid systems.^18–21^ Employing the probe method, we estimate that a fraction of Fock exchange equal to approximately 25% satisfies the generalized Koopmans’ condition for OHT-T. We include non local-electron correlation via the self-consistent rVV10 scheme, with the b parameter determining the extent of non-local van der Waals interaction set it its original value of 6.3. A sizable speed-up of hybrid-DFT calculations is ensured by using the auxiliary matrix method, as implemented in CP2K. In particular, we use the cFIT basis set to calculate the explicit exchange integrals.^22^ Calculations are carried out adopting a supercell approach: we consider a 2x2x1 supercell OHT-T^I^ and a 2x2x3 supercell for OHT-T^II^, both including 768 atoms and corresponding to the experimental density of the materials. The same computational set-up is adopted to perform time-dependent (TD) DFT calculations, as implemented in CP2K, to estimate the optical band gap of the material.^23,24^ In TD-DFT calculations, the criteria for convergence of the excited states is set to 10^-5^ eV. Finally, we use the same supercell to study the energetics of the reaction mechanism leading to the [2+2] photo-cycloaddition reaction in OHT-T materials. To model triplet excited states, we employ the unrestricted Kohn-Sham formalism.

Synthesis

Scheme S1 – Synthesis scheme of OHT-T polymorphs. i) DCM, Et_3_N, CH_3_I, RT; ii) CH_3_I, reflux, 7h; iii) ethanol, piperidine, reflux, 24h; iv) water, sodium tosylate, reflux, 1h, dark; v) 140°C, 3h; vi) ethanol, piperidine, reflux, 24h.

Scheme S2 – Scheme of the [2+2]cycloaddition.

*2-methyl-5-(methylthio)-1,3,4-thiadiazole (****1****).* A mixture of 5-methyl-1,3,4-thiadiazole-2-thiol (4.00 g, 30.2 mmol) and triethylamine (3.06 g, 30.2 mmol) was stirred in 50 mL dichloromethane (50 mL) at room-temperature. Iodomethane (2.00 mL, 32.1 mmol) was then added. After 30 minutes the mixture was poured into *n*-hexane (100mL) under stirring. The precipitated product was filtered to afford 4.37 g of 2-methyl-5-(methylthio)-1,3,4-thiadiazole, yield 99%. ^1^H NMR (500 MHz, [D_6_]DMSO, ppm): δ=2.72 (s, 3H), 2.67 (s, 3H).

*2,3-dimethyl-5-(methylthio)-1,3,4-thiadiazol-3-ium iodide (****2****).* To **1** (4.39 g, 30 mmol), 16 mL of methyl iodide were added and the mixture was refluxed for 7 h. The resulting white product was recovered with acetone, filtered and washed with fresh acetone. The solid was recrystallized in 75 mL of ethanol to afford 3.74 g of **2**, yield 43%. ^1^H NMR (500 MHz, [D_6_]DMSO, ppm) δ=4.15 (s, 3H), 2.97 (s, 3H), 2.77 (s, 3H).^13^C NMR (400 MHz, [D_6_]DMSO, ppm) δ=174.0, 169.5, 41.9, 16.4, 15.0.

*2,3-dimethyl-5-(methylthio)-1,3,4-thiadiazol-3-ium 4-methylbenzenesulfonate (****3****).* A mixture of **1** (1.00 g, 6.84 mmol) and methyl 4-methylbenzenesulfonate (1.65 g, 8.89 mmol) was heated to 140 °C for 3 h. The resulting oil was treated with 17 mL of isopropanol to afford 1.45 g of **3** as a yellow powder, yield 64%. ^1^H NMR (400 MHz, [D_6_]DMSO, ppm) δ=7.47 (d, 2H), 7.11 (d, 2H), 4.14 (s, 3H), 2.96 (s, 3H), 2.77 (s, 3H), 2.29 (s, 3H). ^13^C NMR (400 MHz, [D_6_]DMSO, ppm) δ=174.1, 169.6, 145.8, 137.5, 128.0, 125.5, 41.7, 20.8, 16.3, 14.8.

*(E)-2-(4-hydroxystyryl)-3-methyl-5-(methylthio)-1,3,4-thiadiazol-3-ium iodide (****OHT-I****).* A solution of **2** (1.67 g, 10.3 mmol), 4-hydroxybenzaldehyde (1.26 g, 10.3 mmol) and piperidine (43.8 mg, 0.51 mol) in 25 mL of absolute ethanol was refluxed for 24h and cooled to room temperature. An orange powder was obtained, filtered and washed with cold ethanol to afford 1,33 g of (E)-2-(4-hydroxystyryl)-3-methyl-5-(methylthio)-1,3,4-thiadiazol-3-ium iodide (**OHT-I**), yield 33%. ^1^H NMR (500 MHz, [D_6_]DMSO, ppm) δ=10.56 (s, 1H), 7.93 – 7.82 (m, 3H), 7.54 (d, 1H), 6.91 (d, 2H), 4.25 (s, 3H), 2.81 (s, 3H).^13^C NMR (400 MHz, [D_6_]DMSO, ppm) δ=170.5, 166.8, 162.0, 150.4, 132.2, 125.2, 116.2, 107.5, 41.7, 16.6.

*OHT-T^I^.* To a solution of **OHT-I** (1.33 g, 3.39 mmol) in 100 mL of boiling water, sodium tosylate (2.63 g, 13.6 mmol) was added. After refluxing for 1h in the dark, the resulting solution was cooled to room temperature. Large needle shaped orange crystals were obtained, filtered and washed with cold water to afford 1.20 g of OHT-T^I^, yield 81%. ^1^H NMR (500 MHz, [D_6_]DMSO, ppm) δ=10.58 (s, 1H), 7.92 – 7.81 (m, 3H), 7.54 (d, 1H), 7.47 (d, 2H), 7.10 (d, 2H), 6.91 (d, 2H), 4.25 (s, 3H), 2.81 (s, 3H), 2.28 (s, 2H). ^13^C NMR (400 MHz, [D_6_]DMSO, ppm) δ=170.6, 166.9, 162.0, 150.5, 145.7, 137.6, 132.3, 128.1, 125.5, 125.3, 116.3, 107.5, 41.6, 20.8, 16.5. Prismatic crystals were obtained by slow evaporation of a methanol solution at room temperature.

*OHT-T^II^*. To a solution of **3** (0.257 g, 0.774 mmol) and 4-hydroxybenzaldehyde (0.086 g, 0.704 mmol) dissolved in 10 mL of absolute ethanol, piperidine was added (3.00 mg, 35.2 μmol). After refluxing for 24h, the resulting solution was concentrated under vacuum and 20 mL of chloroform were added. An orange solid precipitated, filtered and washed with cold chloroform to afford 0.030 g of OHT-T (II) (yield 10%). Single crystals were grown by slow evaporation of a methanol/isopropanol solution (1:1) at 40 °C.

*(OHT-T)_2_*. ^1^H NMR (400 MHz, [D_6_]DMSO, ppm) δ=9.76 (s, 2H), 7.48 (d, 4H), 7.37 (d, 4H), 7.11 (d, 4H), 6.79 (d, 4H), 5.23 (t, 2H), 4.92 (t, 2H), 3.95 (s, 6H), 2.70 (s, 6H), 2.29 (s, 6H).

*Light penetration depth in OHT-T^I^*. The transmittance profile across OHT-T^I^ crystals was calculated using the Lambert-Beer law:

$$T=\frac{I}{I_{0}}=e^{-\alpha_{\lambda}\cdot l}$$

Where *α_λ_* is the absorption coefficient of the crystal and *l* is the optical path. The molar absorption coefficient ε_λ_ of OHT-T was measured in solution at the three wavelengths corresponding to the emission maxima of the violet, white and blue LEDs used in the PE experiments (400, 444 and 450 nm, respectively). Then, the values of *ε_λ_* were converted to the linear absorption coefficients *α_λ_* according to the formula:^25,26^

$$\alpha_{\lambda}=\frac{1000\cdot\ln\left( 10 \right)\cdot\epsilon_{\lambda}\cdot\rho}{M}$$

$$\alpha_{\lambda}\approx\frac{2303\cdot\epsilon_{\lambda}\cdot\rho}{M}$$

Where ρ is the crystallographic density of OHT-T^I^ (1.4255 g/cm^3^) and M is molar mass of OHT-T (436.56 g/mol).

| λ (nm) | ε/10^4^$\text{(L mol}^{-1} \text{cm}^{-1})$ | α /10^4^ (cm^-1^) |
| --- | --- | --- |
| 400 | 3.080 | 23.2 |
| 444 | 0.484 | 3.64 |
| 450 | 0.205 | 1.54 |


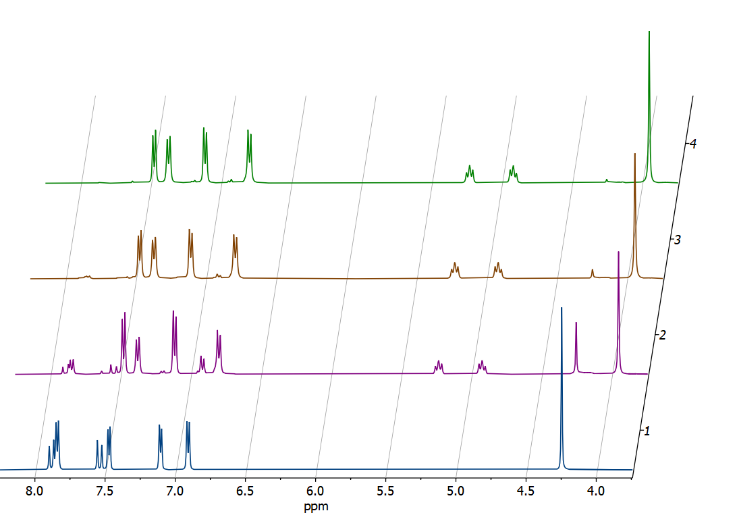

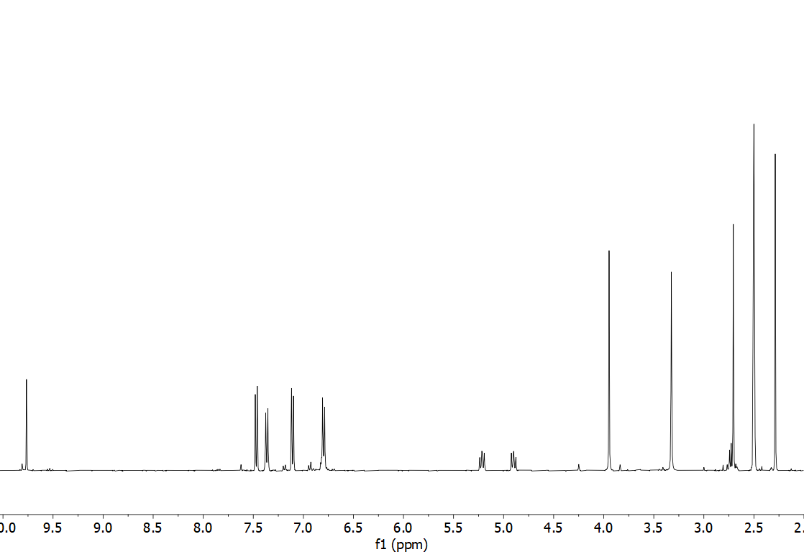


b)

a)

Figure S1 – ^1^H NMR spectra of OHT-T^I^ (a) after irradiation with white light for 0 h (*1*), 5 h (*2*), 10 h (*3*) and 20 h (*4*); ^1^H NMR spectra of OHT-T^II^ (b) after irradiation with white light for 24 h. The estimated dimerization yield was 98% and 97% for OHT-T^I^ and OHT-T^II^, respectively.


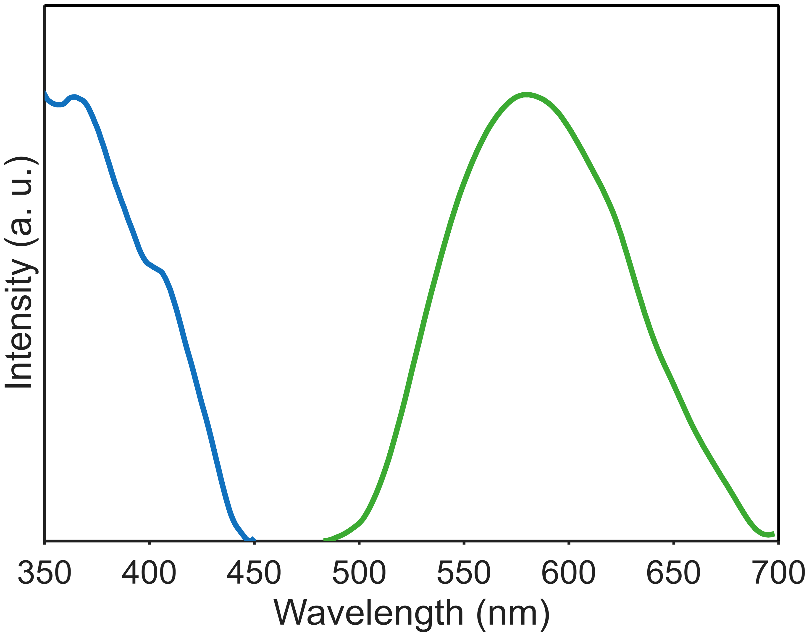


Figure S2 – Excitation (absorption) and emission spectra of OHT-T^II^ (blue and green line, respectively).


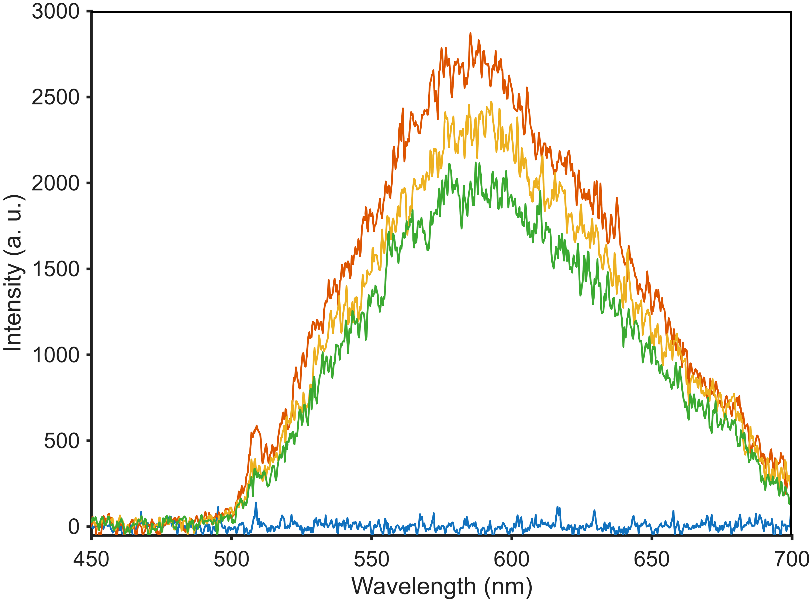


Figure S3 – Fluorescence spectra of OHT-T^I^ under 1030 nm laser exposure after 0 min (red line), 20 min (yellow line), 30 min (green line); fluorescence spectrum of (OHT-T)_2_ (blue line).


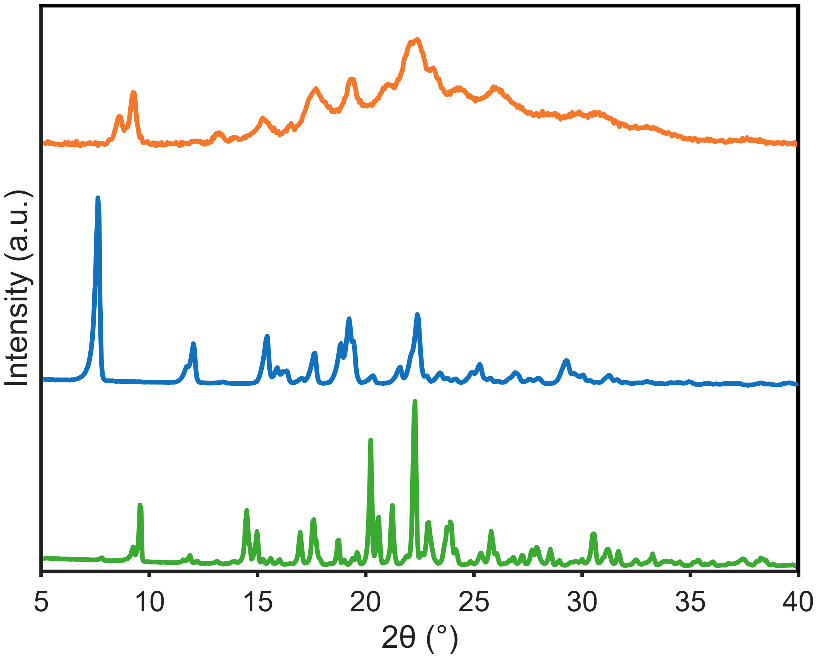


Figure S4 – PXRD pattern of (OHT-T)_2_^I^ (green line), (OHT-T)_2_^II^ (orange line) and (OHT-T)_2_^III^ (blue line), the latter obtained after recrystallization from anhydrous dimethyl sulfoxide and acetone.

Table S1 - Crystal data and structure refinement details for OHT-T^I^, OHT-T^II^ and (OHT-T)_2_^III^.

|  | OHT-T^I^ | OHT-T^II^ | (OHT-T)_2_^III^ |
| --- | --- | --- | --- |
| CCDC number | 2526788 | 2526605 | 2526789 |
| Empirical formula | C_19_H_20_N_2_O_4_S_3_ | C_19_H_20_N_2_O_4_S_3_ | C_38_H_40_N_4_O_8_S_6_ |
| Formula weight | 436.56 | 436.56 | 873.12 |
| T (K) | 298.00 | 293(2) | 150.00 |
| λ (Å) | 0.71073 | 0.71073 | 0.71073 |
| Crystal system | Monoclinic | Triclinic | Tetragonal |
| Space group | P2_1_/n | *P-1* | P4_2_/n |
| *a* (Å) | 14.0696(18) | 7.3190(18) | 23.184(2) |
| *b* (Å) | 7.4667(9) | 10.623(5) | 23.184(2) |
| *c* (Å) | 20.108(3) | 14.348(7) | 7.9596(10) |
| *α* (°) | 90 | 69.68(3) | 90 |
| *β* (°) | 106.324(4) | 76.68(3) | 90 |
| *γ* (°) | 90 | 89.82(3) | 90 |
| V (Å^3^) | 2027.3(4) | 1014.4(7) | 4278.3(10) |
| Z | 4 | 2 | 4 |
| D_calc_ (Mg/m^3^) | 1.430 | 1.429 | 1.355 |
| *μ* (mm^-1^) | 0.394 | 0.394 | 0.373 |
| F(000) | 914.0 | 456.0 | 1824.0 |
| Θ range (°) | 2.07 – 26.4 | 2.87 - 27.50 | 2.48 – 25.08 |
| Reflections collected / unique [R(int)] | 27445/4149 [0.0455] | 14465/4536 [0.0600] | 54198/3809 [0.2143] |
| Data/restraints/parameters | 4149/0/377 | 4536/0/259 | 3809/0/285 |
| Goodness-of-fit on F^2^ | 1.060 | 1.046 | 1.015 |
| Final*R1*, *wR2* indices [I>2*σ*(I)] | 0.0273, 0.0606 | 0.0623, 0.1484 | 0.0666, 0.1463 |
| Final *R1*, *wR2* indices (all data) | 0.0364, 0.0660 | 0.0978, 0.1745 | 0.1583, 0.1973 |
| Largest diff. peak / hole (eÅ^-3^) | 0.19/-0.18 | 0.54/-0.33 | 0.38/-0.48 |


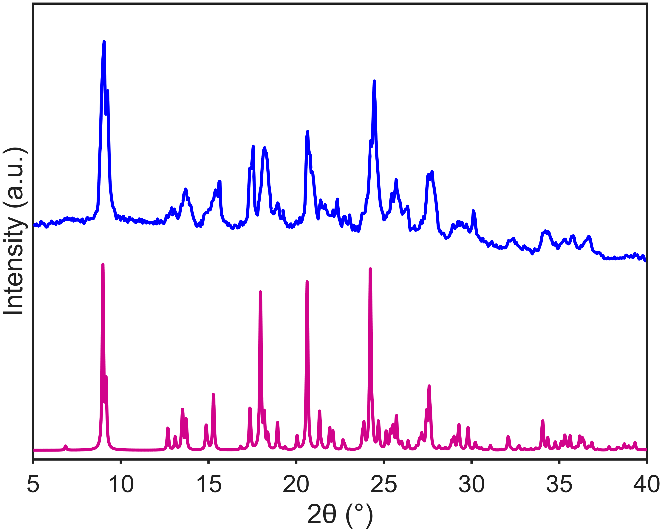


Figure S5 – Experimental (blue line) and calculated (red line) PXRD pattern of OHT-T^I^.


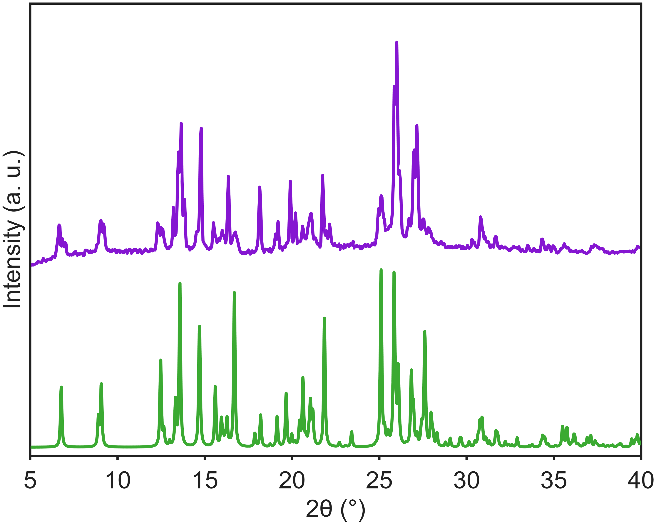


Figure S6 – Experimental (purple line) and calculated (green line) PXRD pattern of OHT-T^II^.


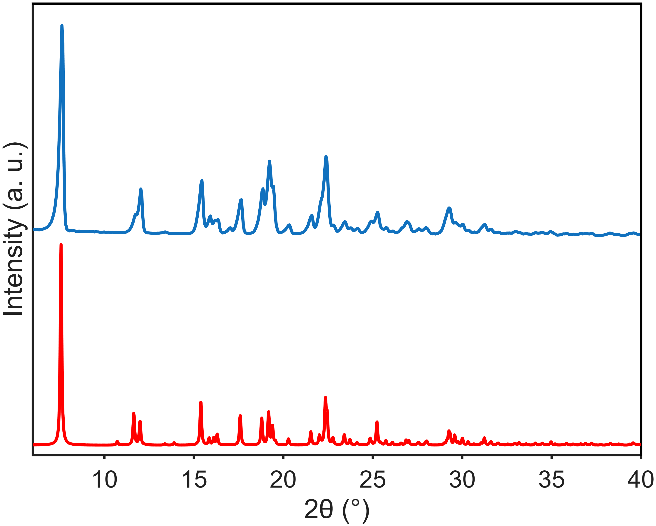


Figure S7 – Experimental (blue line) and calculated (red line) PXRD pattern of (OHT-T)_2_^III^.


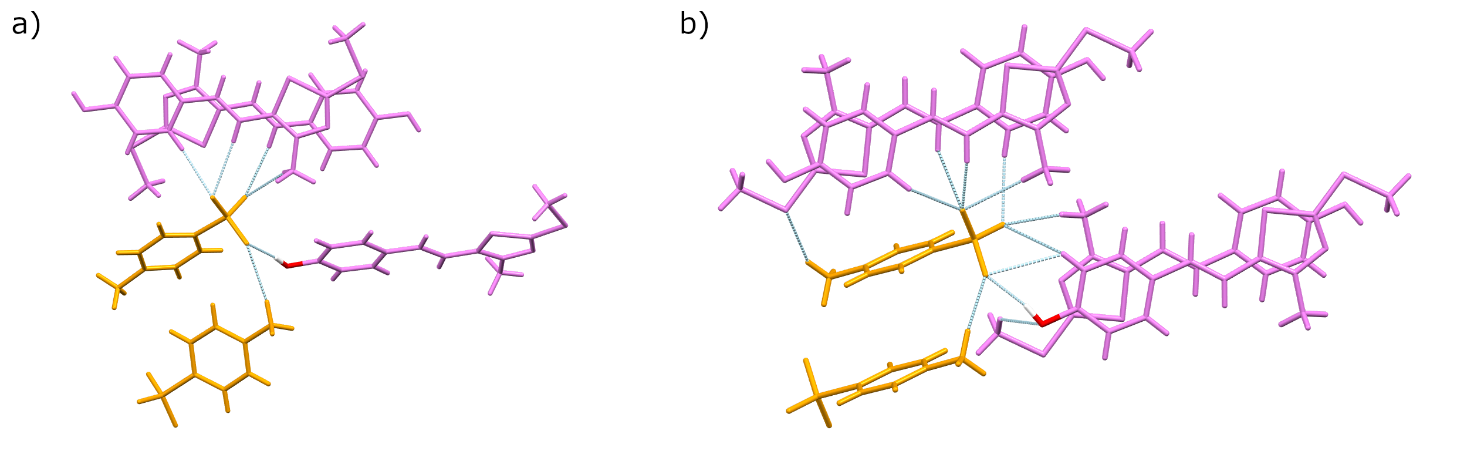


Figure S8 – Detail of crystal packing of OHT-T^I^ (a) and of OHT-T^II^ (b) with strong and weak hydrogen bonds highlighted.


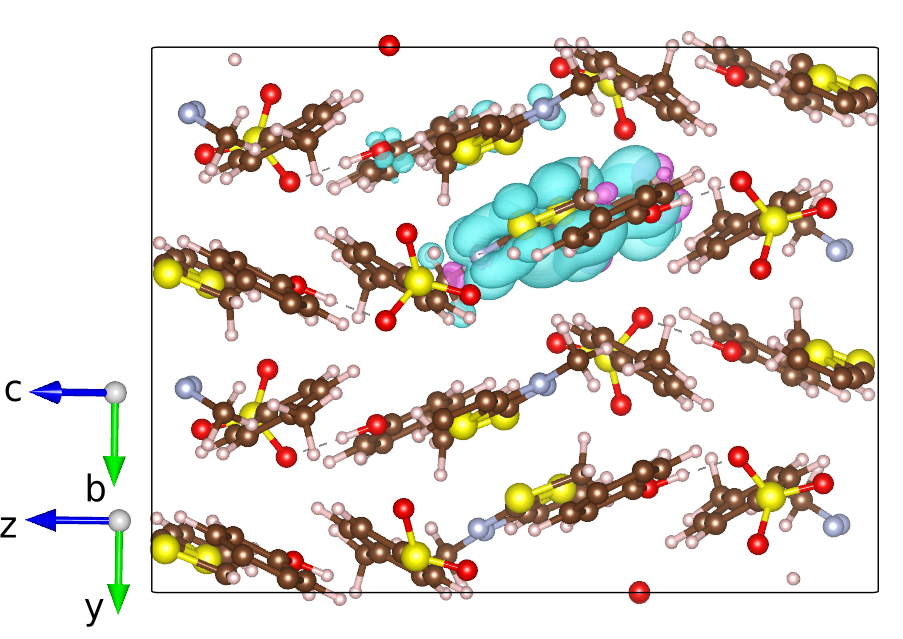


Figure S9. Isosurface contour plot of the spin density of the most stable triplet state after geometry relaxation. Atom color code: C brown, H white, N blue, O red, S yellow.


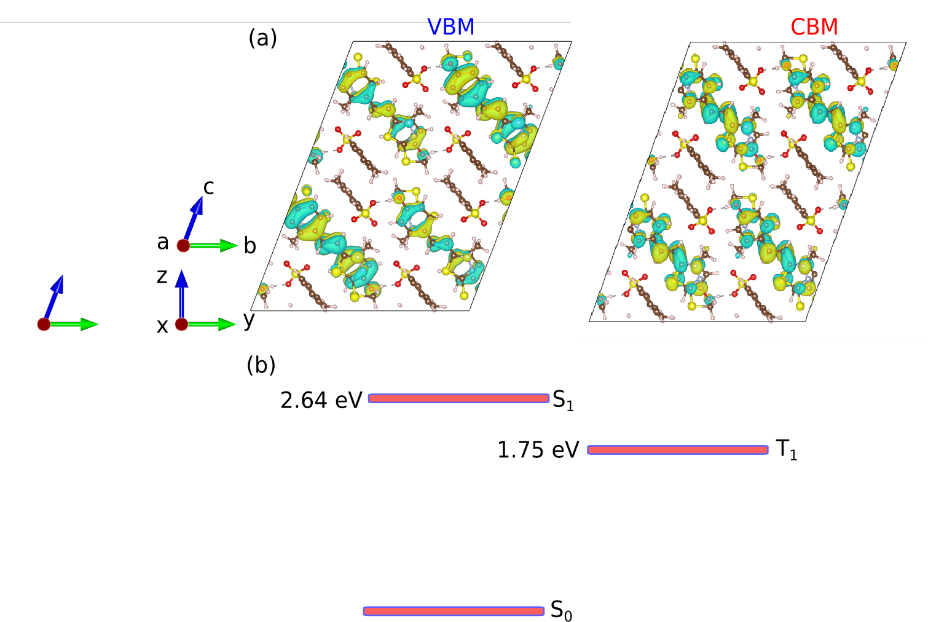


Figure S10. (b) Isodensity representation of the valence band maximum and of the conduction band minimum for OHT-T^II^. In the representation, the *b* lattice vectors points outwards. C atoms in brown, H in white, N in blue, O in red, S in yellow. (b) Jablonski diagram of OHT-T^II^.


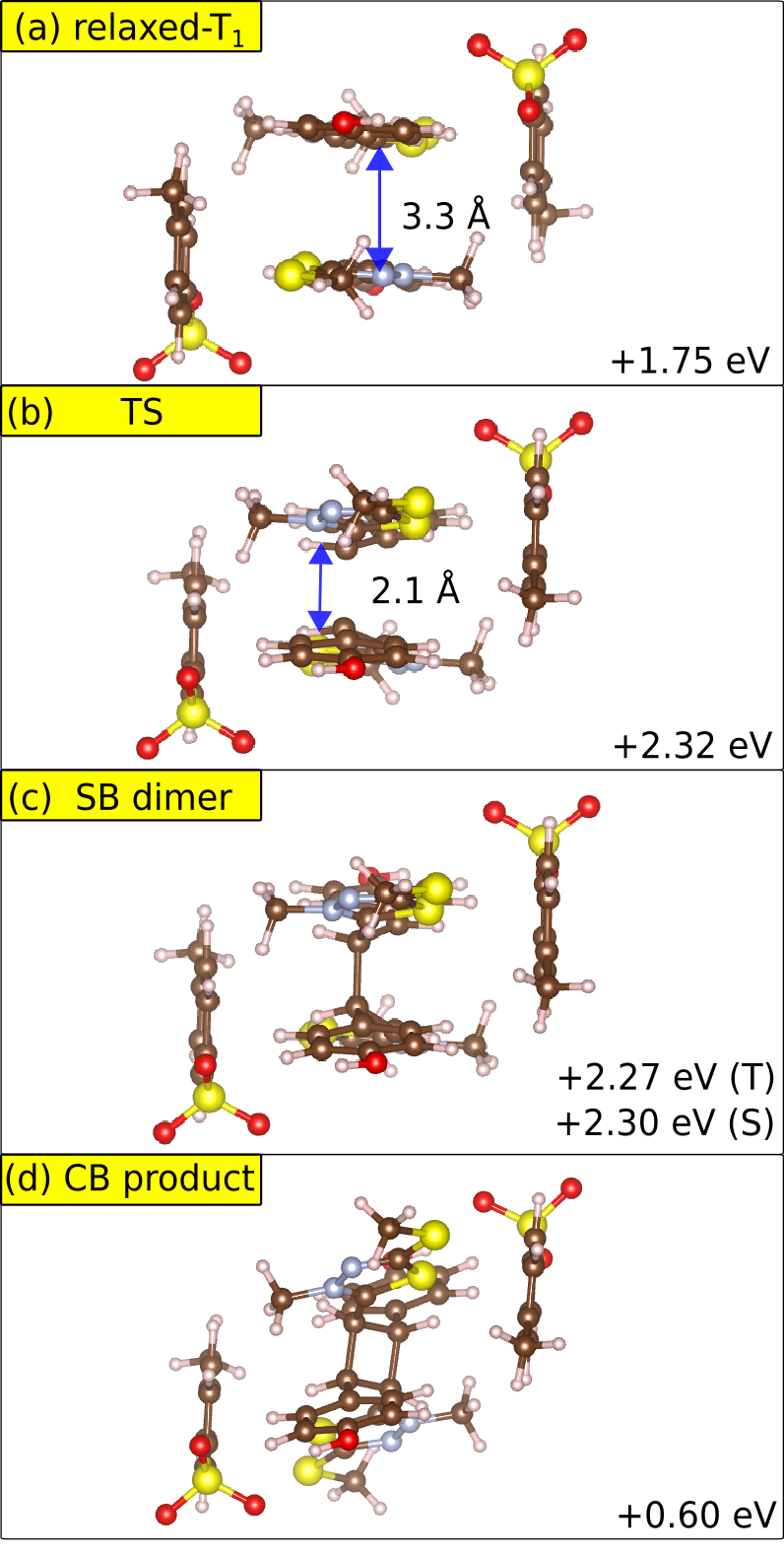


Figure S11. Structural representation of (a) relaxed triplet state (r-T_1_) (b) transition state (TS), (c) singly bound dimer (SB) and (d) cyclobutane (CB) product in bulk OHT-T^II^, cf. main text for complete description. For clarity of representation, only the stilbene units involved in the reaction and the closest counter-ions are depicted in the figure. C atoms in brown, H in white, N in blue, O in red, S in yellow. For each structural configuration, we also report the respective total energy, as referred to that of the supercell of the pristine material in the ground state. For the SB dimer, we include the energy for both the triplet (T) and the singlet (S) states.


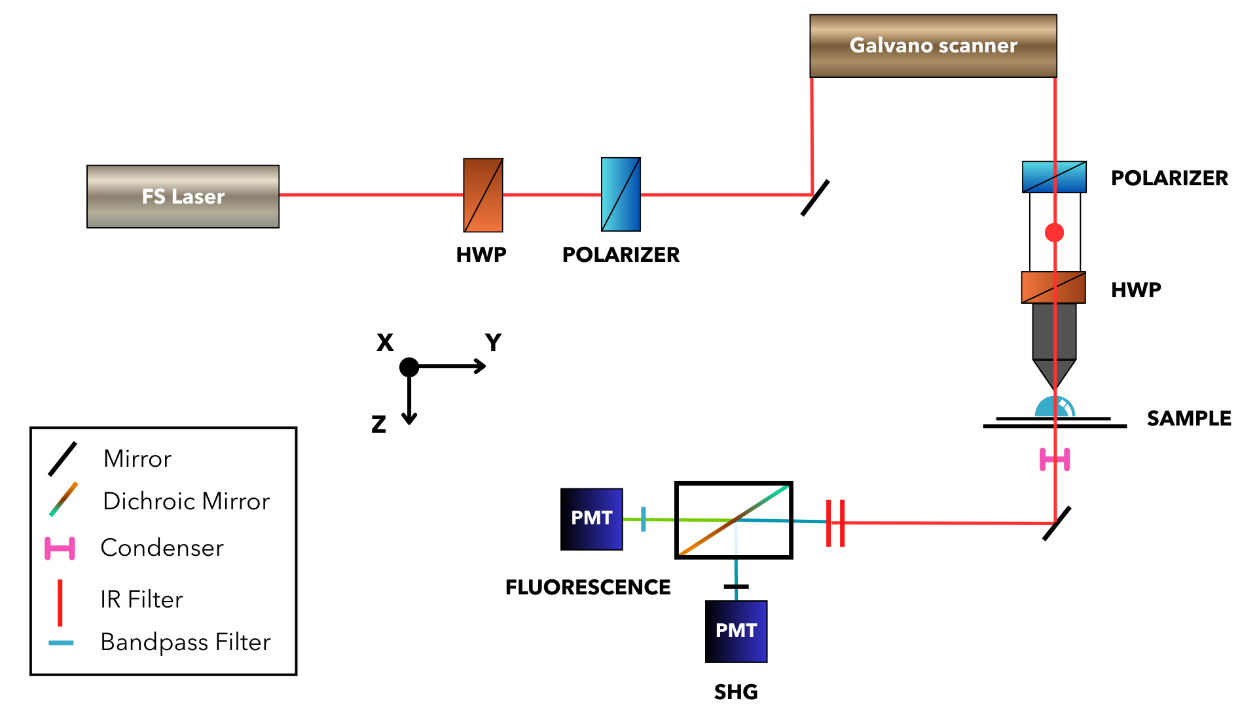


Figure S12. Schematic representation of the experimental setup, along with a laboratory coordinate system.


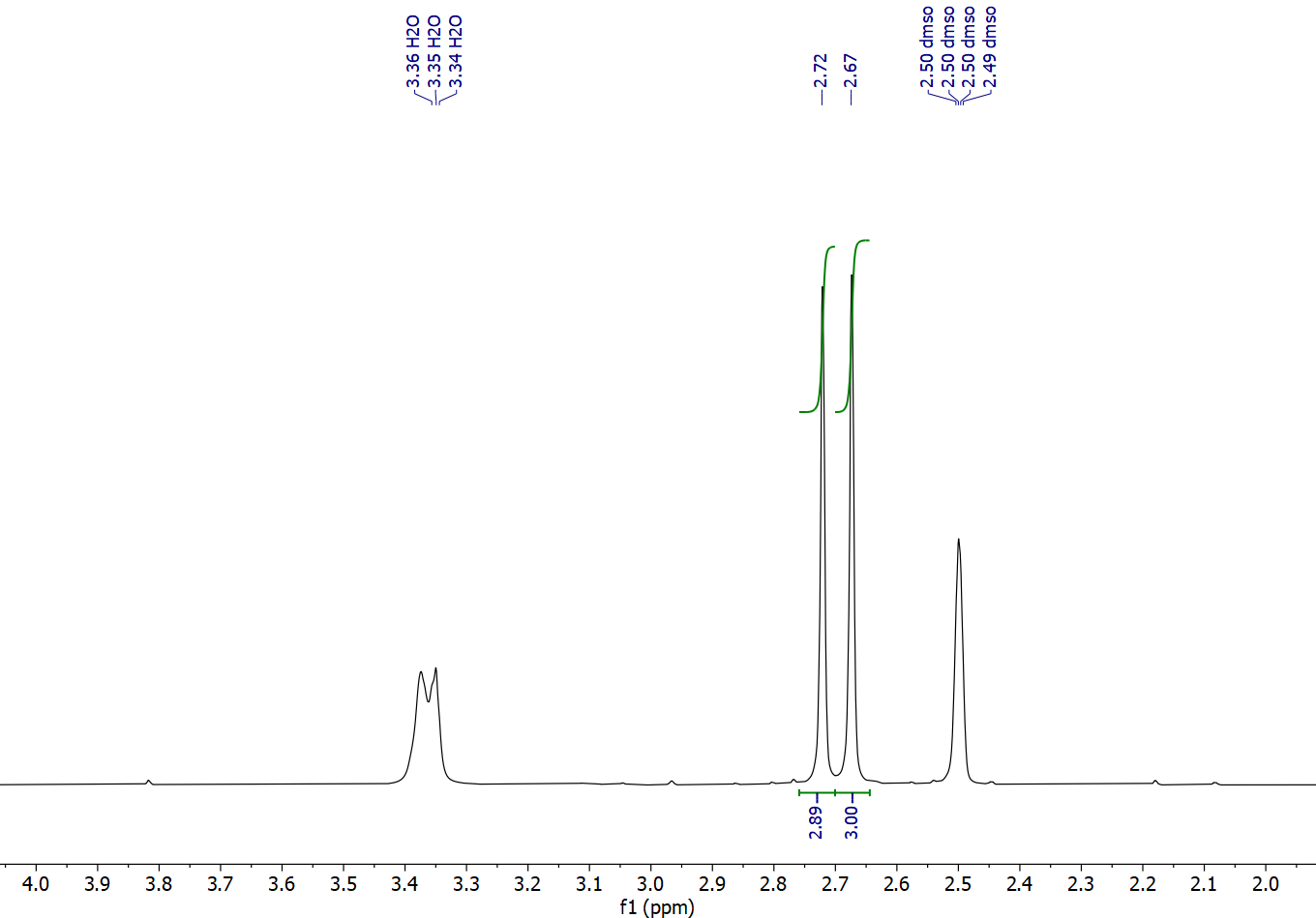


Figure S13. ^1^H NMR spectrum of 2-methyl-5-(methylthio)-1,3,4-thiadiazole.


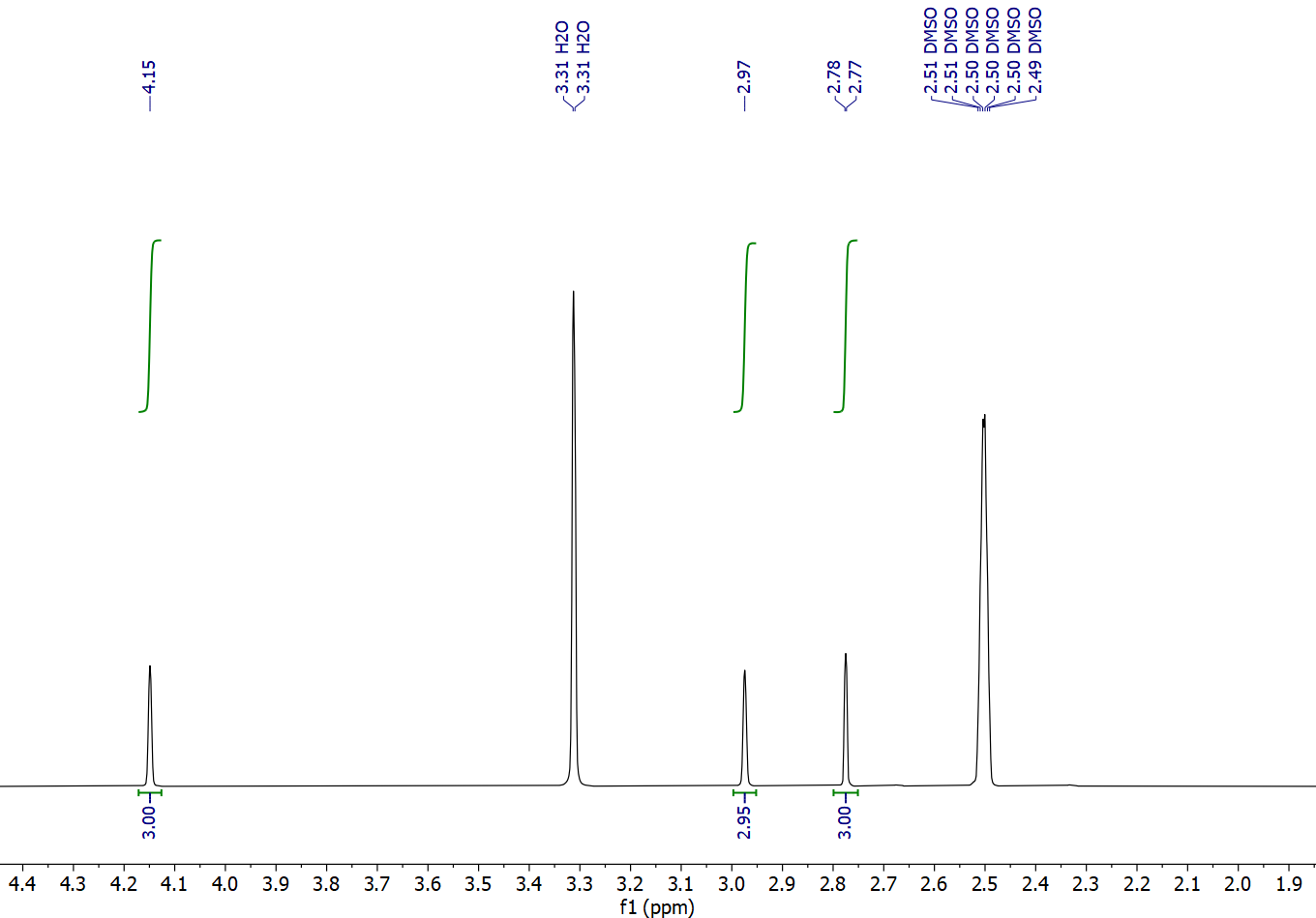


Figure S14. ^1^H NMR spectrum of 2,3-dimethyl-5-(methylthio)-1,3,4-thiadiazol-3-ium iodide.


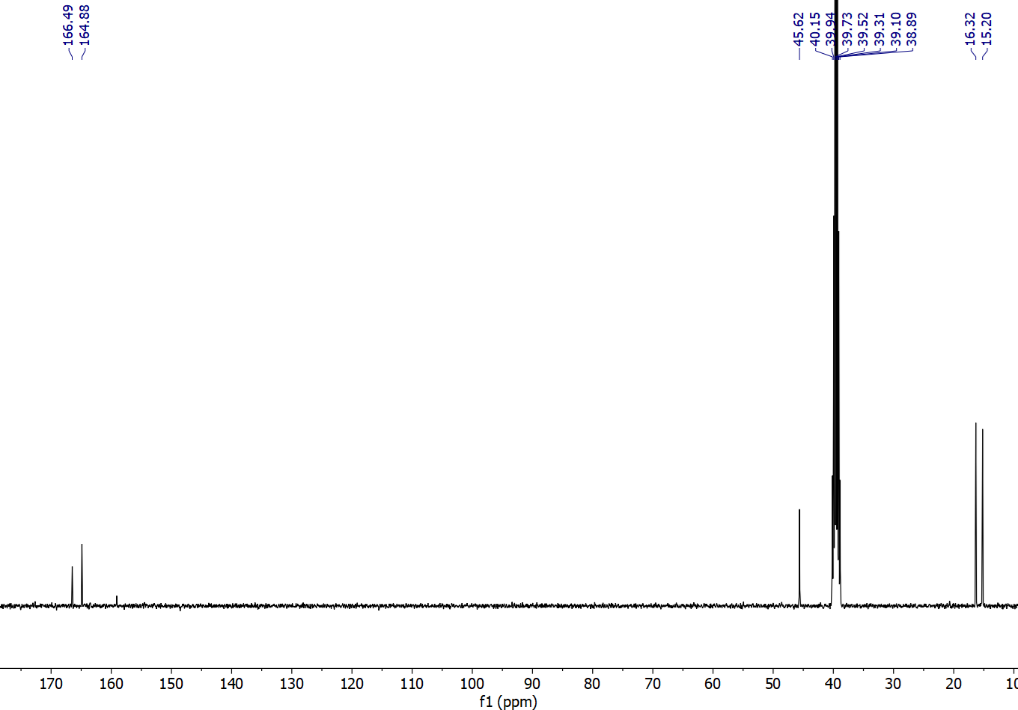


Figure S15. ^13^C NMR spectrum of 2,3-dimethyl-5-(methylthio)-1,3,4-thiadiazol-3-ium iodide.

*
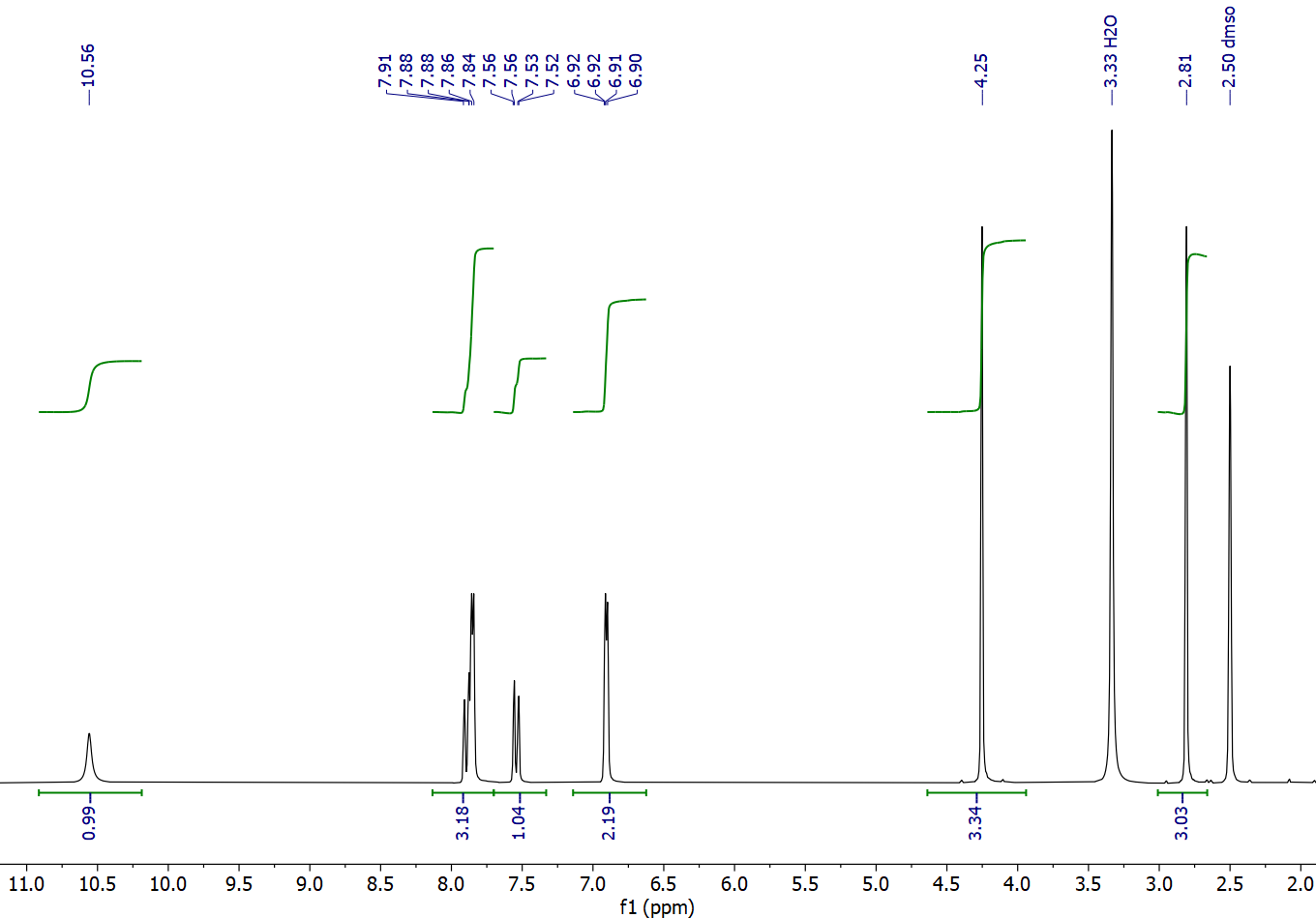
*

Figure S16. ^1^H NMR spectrum of (E)-2-(4-hydroxystyryl)-3-methyl-5-(methylthio)-1,3,4-thiadiazol-3-ium iodide (OHT-I).


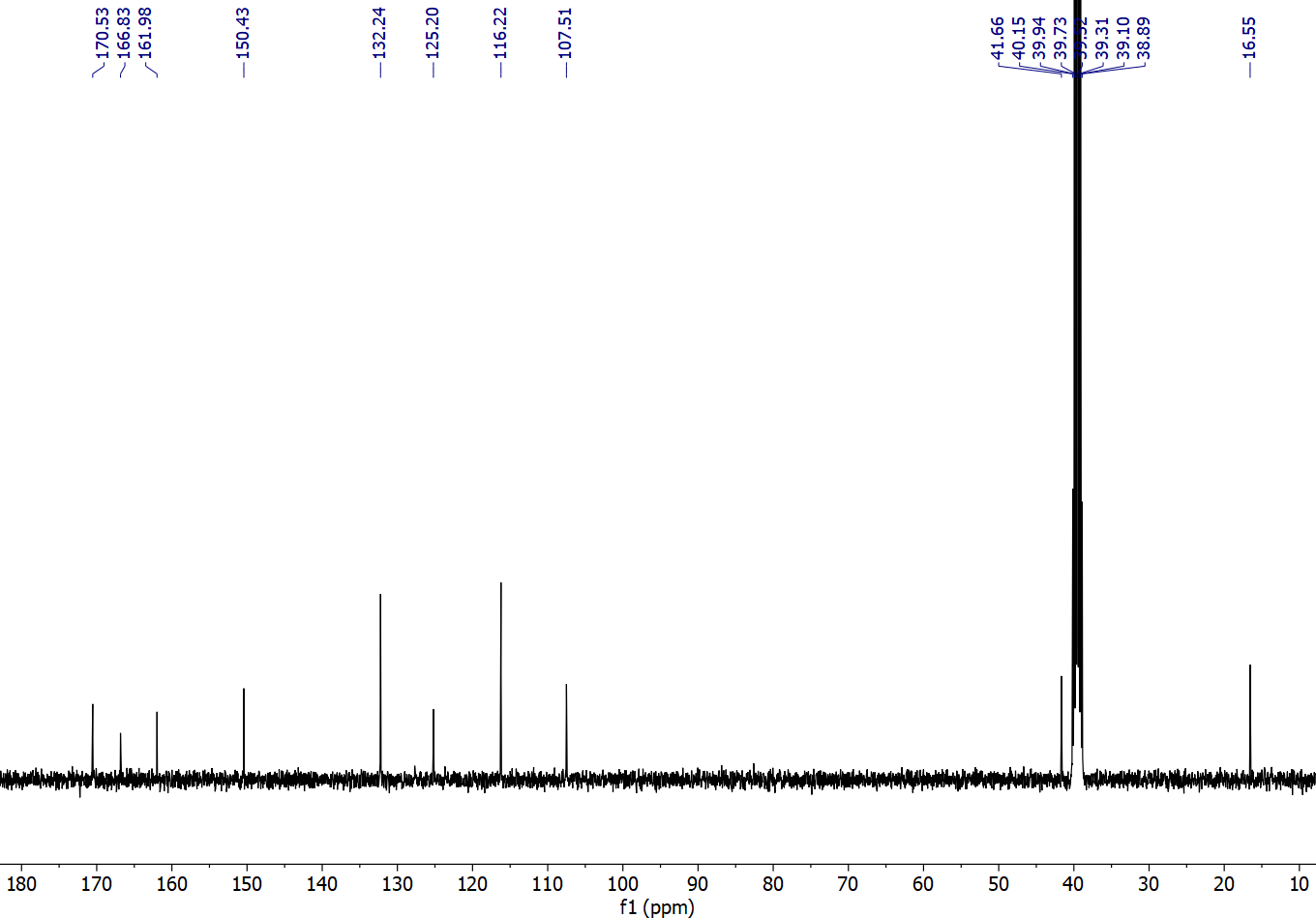


Figure S17. ^13^C NMR spectrum of (E)-2-(4-hydroxystyryl)-3-methyl-5-(methylthio)-1,3,4-thiadiazol-3-ium iodide (OHT-I).

*
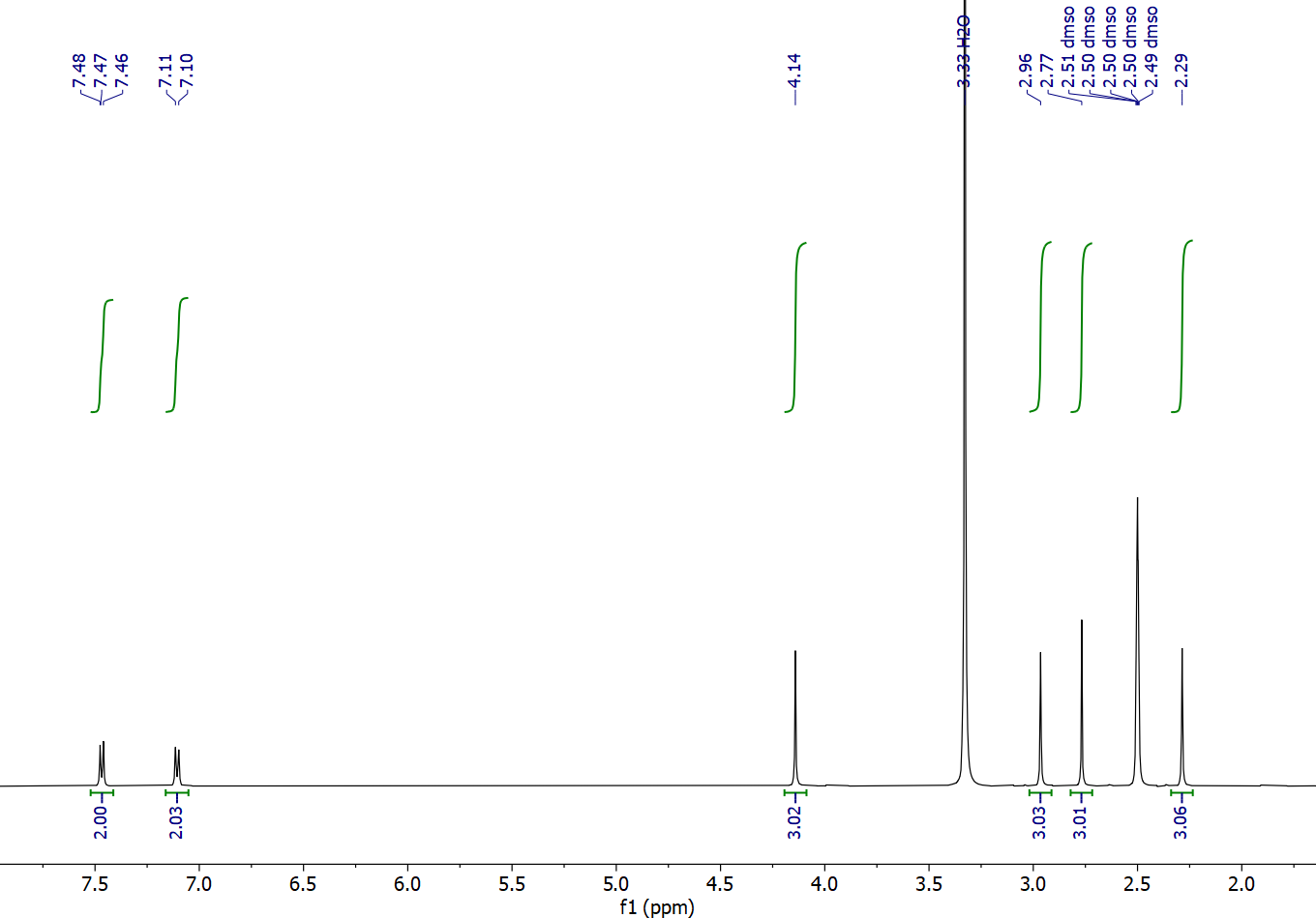
*

Figure S18. ^1^H NMR spectra of 2,3-dimethyl-5-(methylthio)-1,3,4-thiadiazol-3-ium 4-methylbenzenesulfonate


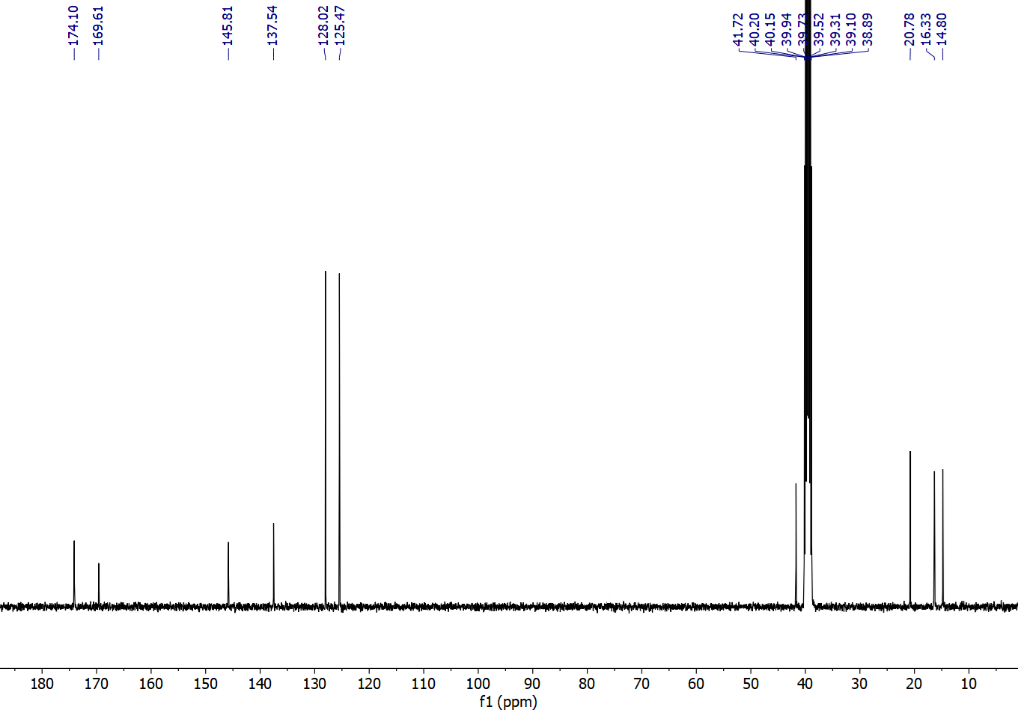


Figure S19. ^13^C NMR spectra of 2,3-dimethyl-5-(methylthio)-1,3,4-thiadiazol-3-ium 4-methylbenzenesulfonate


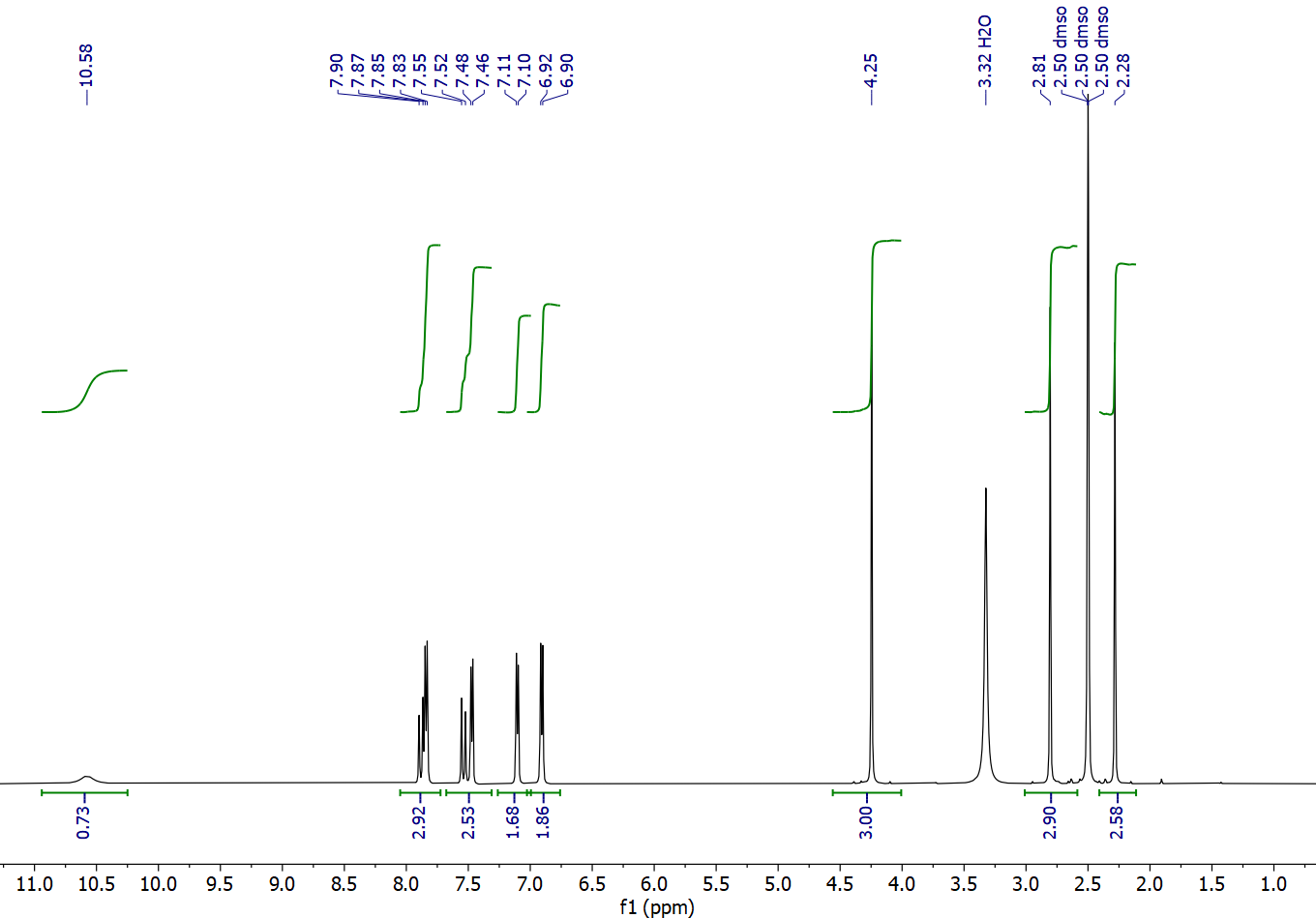


Figure S20. ^1^H NMR spectra of (E)-2-(4-hydroxystyryl)-3-methyl-5-(methylthio)-1,3,4-thiadiazol-3-ium 4-methylbenzenesulfonate (OHT-T).


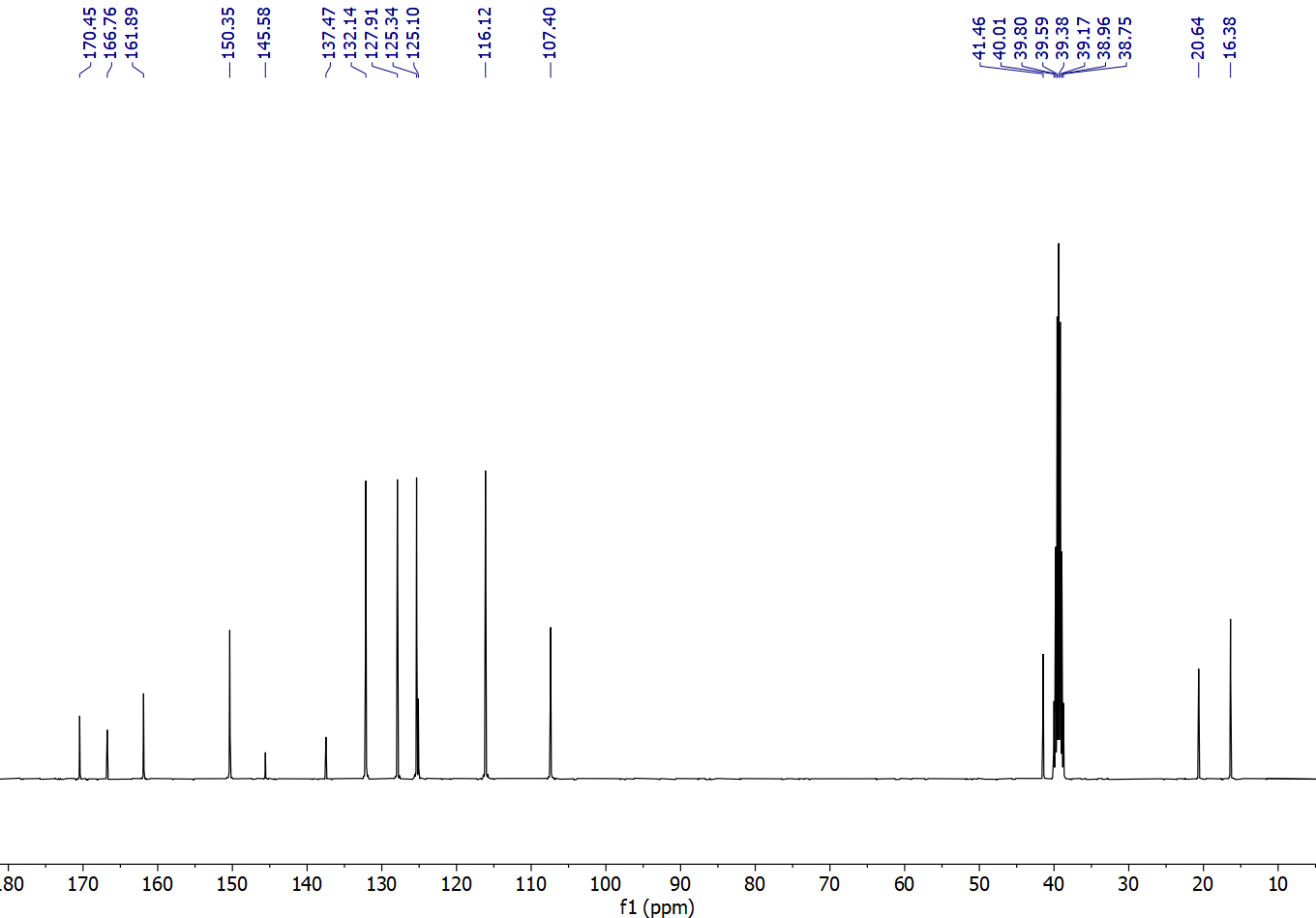


Figure S21. ^13^C NMR spectra of (E)-2-(4-hydroxystyryl)-3-methyl-5-(methylthio)-1,3,4-thiadiazol-3-ium 4-methylbenzenesulfonate (OHT-T).

**References**

[1] G. M. Sheldrick, “Crystal structure refinement with SHELXL” *Acta Crystallogr. Sect. C Struct. Chem.* **2015**, *71*, 3–8, https://doi.org/10.1107/S2053229614024218.

[2] K. K. Jha, B. Gruza, P. Kumar, M. L. Chodkiewicz, P. M. Dominiak, “TAAM: a reliable and user friendly tool for hydrogen-atom location using routine X-ray diffraction data” *Acta Crystallogr. Sect. B Struct. Sci. Cryst. Eng. Mater.* **2020**, *76*, 296–306, https://doi.org/10.1107/S2052520620002917.

[3] N. K. Hansen, P. Coppens, “Testing aspherical atom refinements on small-molecule data sets” *Acta Crystallogr. Sect. A* **1978**, *34*, 909–921, https://doi.org/10.1107/S0567739478001886.

[4] O. V. Dolomanov, L. J. Bourhis, R. J. Gildea, J. A. K. Howard, H. Puschmann, “OLEX2: A complete structure solution, refinement and analysis program” *J. Appl. Crystallogr.* **2009**, *42*, 339–341, https://doi.org/10.1107/S0021889808042726.

[5] F. Kleemiss, O. V. Dolomanov, M. Bodensteiner, N. Peyerimhoff, L. Midgley, L. J. Bourhis, A. Genoni, L. A. Malaspina, D. Jayatilaka, J. L. Spencer, F. White, B. Grundkötter-Stock, S. Steinhauer, D. Lentz, H. Puschmann, S. Grabowsky, “Accurate crystal structures and chemical properties from NoSpherA2” *Chem. Sci.* **2021**, *12*, 1675–1692, https://doi.org/10.1039/D0SC05526C.

[6] M. L. Chodkiewicz, S. Migacz, W. Rudnicki, A. Makal, J. A. Kalinowski, N. W. Moriarty, R. W. Grosse-Kunstleve, P. V. Afonine, P. D. Adams, P. M. Dominiak, “DiSCaMB: a software library for aspherical atom model X-ray scattering factor calculations with CPUs and GPUs” *J. Appl. Crystallogr.* **2018**, *51*, 193–199, https://doi.org/10.1107/S1600576717015825.

[7] K. K. Jha, B. Gruza, A. Sypko, P. Kumar, M. L. Chodkiewicz, P. M. Dominiak, “Multipolar atom types from theory and statistical clustering (MATTS) data bank: restructurization and extension of UBDB” *J. Chem. Inf. Model.* **2022**, *62*, 3752–3765, https://doi.org/10.1021/acs.jcim.2c00144.

[8] A. Altomare, M. C. Burla, M. Camalli, G. L. Cascarano, C. Giacovazzo, A. Guagliardi, A. G. G. Moliterni, G. Polidori, R. Spagna, “SIR 97: a new tool for crystal structure determination and refinement” *J. Appl. Crystallogr.* **1999**, *32*, 115–119, https://doi.org/10.1107/S0021889898007717.

[9] G. M. Sheldrick, “Crystal structure refinement with SHELXL” *Acta Crystallogr. Sect. C Struct. Chem.* **2015**, *71*, 3–8, https://doi.org/10.1107/S2053229614024218.

[10] C. F. Macrae, I. Sovago, S. J. Cottrell, P. T. A. Galek, P. McCabe, E. Pidcock, M. Platings, G. P. Shields, J. S. Stevens, M. Towler, P. A. Wood, “Mercury 4.0: from visualization to analysis, design and prediction” *J. Appl. Crystallogr.* **2020**, *53*, 226–235, https://doi.org/10.1107/S1600576719014092.

[11] J. VandeVondele, M. Krack, F. Mohamed, M. Parrinello, T. Chassaing, J. Hutter, “Quickstep: fast and accurate density functional calculations using a mixed gaussian and plane waves approach” *Comput. Phys. Commun.* **2005**, *167*, 103–128, https://doi.org/10.1016/j.cpc.2004.12.014.

[12] J. VandeVondele, J. Hutter, “Gaussian basis sets for accurate calculations on molecular systems in gas and condensed phases” *J. Chem. Phys.* **2007**, *127*, 114105, https://doi.org/10.1063/1.2770708.

[13] S. Goedecker, M. Teter, J. Hutter, “Separable dual-space gaussian pseudopotentials” *Phys. Rev. B* **1996**, *54*, 1703–1710, https://doi.org/10.1103/PhysRevB.54.1703.

[14] C. Adamo, V. Barone, “Toward reliable density functional methods without adjustable parameters: the PBE0 model” *J. Chem. Phys.* **1999**, *110*, 6158–6170, https://doi.org/10.1063/1.478522.

[15] G. Miceli, W. Chen, I. Reshetnyak, A. Pasquarello, “Nonempirical hybrid functionals for band gaps and polaronic distortions in solids” *Phys. Rev. B* **2018**, *97*, 121112, https://doi.org/10.1103/PhysRevB.97.121112.

[16] T. Bischoff, I. Reshetnyak, A. Pasquarello, “Adjustable potential probes for band-gap predictions of extended systems through nonempirical hybrid functionals” *Phys. Rev. B* **2019**, *99*, 201114, https://doi.org/10.1103/PhysRevB.99.201114.

[17] T. Bischoff, J. Wiktor, W. Chen, A. Pasquarello, “Nonempirical hybrid functionals for band gaps of inorganic metal-halide perovskites” *Phys. Rev. Mater.* **2019**, *3*, 123802, https://doi.org/10.1103/PhysRevMaterials.3.123802.

[18] F. Ambrosio, A. Landi, M. Loriso, A. Leo, A. Peluso, “External reorganization energy upon charge transfer reactions in mildly polar media: the case of naphthalene in tetrahydrofuran” *J. Phys. Chem. Lett.* **2025**, *16*, 6734–6744, https://doi.org/10.1021/acs.jpclett.5c01328.

[19] F. Ambrosio, J. Wiktor, A. Landi, A. Peluso, “Charge localization in acene crystals from *ab initio* electronic structure” *J. Phys. Chem. Lett.* **2023**, *14*, 3343–3351, https://doi.org/10.1021/acs.jpclett.3c00191.

[20] F. Ambrosio, A. Capobianco, A. Landi, T. Pizza, A. Peluso, “Is a thin mechanism appropriate for aromatic nitration?” *Phys. Chem. Chem. Phys.* **2023**, *25*, 2359–2365, https://doi.org/10.1039/D2CP05176A.

[21] F. Ambrosio, A. Landi, A. Peluso, A. Capobianco, “Quantum chemical insights into DNA nucleobase oxidation: bridging theory and experiment” *J. Chem. Theory Comput.* **2024**, *20*, 9708–9719, https://doi.org/10.1021/acs.jctc.4c01045.

[22] M. Guidon, J. Hutter, J. VandeVondele, “Auxiliary density matrix methods for hartree-fock exchange calculations” *J. Chem. Theory Comput.* **2010**, *6*, 2348–2364, https://doi.org/10.1021/ct1002225.

[23] J. Strand, S. K. Chulkov, M. B. Watkins, A. L. Shluger, “First principles calculations of optical properties for oxygen vacancies in binary metal oxides” *J. Chem. Phys.* **2019**, *150*, 044702, https://doi.org/10.1063/1.5078682.

[24] M. Iannuzzi, T. Chassaing, T. Wallman, J. Hutter, “Ground and excited state density functional calculations with the gaussian and augmented-plane-wave method” *Chimia* **2005**, *59*, 499–499, https://doi.org/10.2533/000942905777676164.

[25] “B. Valeur 2001, Chapter 2-Absorption of UV–Visible Light.” In Molecular fluorescence: principles and applications. Wiley-VCH Verlag GmbH. ISBN 978-3-527-60024-3.

[26] J. R. Lakowicz, Ed. , *Principles of fluorescence spectroscopy*, Springer US, Boston, MA, **2006**.
